# Delineating a Conserved Genetic Cassette Promoting Outgrowth of Body Appendages

**DOI:** 10.1371/journal.pgen.1003231

**Published:** 2013-01-24

**Authors:** Congxing Lin, Yan Yin, Sheila M. Bell, G. Michael Veith, Hong Chen, Sung-Ho Huh, David M. Ornitz, Liang Ma

**Affiliations:** 1 Division of Dermatology, Department of Medicine, Washington University School of Medicine, St. Louis, Missouri, United States of America; 2 Perinatal Institute of Cincinnati Children's Hospital Medical Center, Division of Neonatology-and Pulmonary Biology, University of Cincinnati College of Medicine, Cincinnati, Ohio, United States of America; 3 Department of Developmental Biology, Washington University School of Medicine, St. Louis, Missouri, United States of America; Harvard Medical School, United States of America

## Abstract

The acquisition of the external genitalia allowed mammals to cope with terrestrial-specific reproductive needs for internal fertilization, and thus it represents one of the most fundamental steps in evolution towards a life on land. How genitalia evolved remains obscure, and the key to understanding this process may lie in the developmental genetics that underpins the early establishment of the genital primordium, the genital tubercle (GT). Development of the GT is similar to that of the limb, which requires precise regulation from a distal signaling epithelium. However, whether outgrowth of the GT and limbs is mediated by common instructive signals remains unknown. In this study, we used comprehensive genetic approaches to interrogate the signaling cascade involved in GT formation in comparison with limb formation. We demonstrate that the FGF ligand responsible for GT development is FGF8 expressed in the cloacal endoderm. We further showed that forced *Fgf8* expression can rescue limb and GT reduction in embryos deficient in WNT signaling activity. Our studies show that the regulation of *Fgf8* by the canonical WNT signaling pathway is mediated in part by the transcription factor SP8. *Sp8* mutants elicit appendage defects mirroring WNT and FGF mutants, and abolishing *Sp8* attenuates ectopic appendage development caused by a gain-of-function β-catenin mutation. These observations indicate that a conserved WNT-SP8-FGF8 genetic cassette is employed by both appendages for promoting outgrowth, and suggest a deep homology shared by the limb and external genitalia.

## Author Summary

Mammalian limbs and external genitalia are body appendages specialized for locomotion and internal fertilization, respectively. Despite their marked anatomical and functional differences, development of the limb and external genitalia appears to involve similar genetic controls, and some have suggested that regulatory mechanisms common to both might be evolutionarily linked. One essential aspect for appendage development is the establishment and maintenance of a separated proximodistal developmental axis apart from the main body axis, which is often instructed by a distal signaling epithelium. Herein, we adopted comprehensive mouse genetic approaches to investigate regulatory mechanisms underlying the distal signaling center in the limb and the GT, and uncovered a conserved genetic cassette that is utilized by both paired and unpaired appendages to establish a distal signaling center in the epithelium that mediates subsequent proximodistal outgrowth. Our results further suggested that the evolution of the external genital organ involved co-option of the same genetic program underpinning limb development.

## Introduction

Development of the external genitalia is a crucial aspect of mammalian evolution that enables internal fertilization, a pivotal step towards land invasion. All therian mammals including metatherians develop external genitalia around their urogenital outlets. In mice, development of the embryonic anlage of external genitalia, the genital tubercle (GT), is identical in both sexes before androgen-mediated penile masculinization which occurs around embryonic day 16. The early androgen-independent phase of GT development is achieved through coordinated growth and patterning of cloacal endoderm-derived urethral epithelium (UE), mesoderm-derived para-cloacal mesenchyme (PCM) and the ventral ectoderm, which results in a cone-like structure with a ventral-medial positioned urethra surrounded by GT mesenchyme within an ectodermal epithelial capsule.

The development of the GT as an unpaired body appendage, is often compared to that of the paired-type appendages, the limbs [1], [2]. Despite their anatomical and functional differences, the morphogenesis of both structures appears to involve similar genetic controls. Regulatory genes/pathways including canonical WNT signaling [3], [4], HH signaling [5]–[7], BMP signaling [8], [9] and *Hox* genes [10]–[14] are essential for the development of both appendages. Some have suggested that the regulatory mechanisms common to both might be evolutionarily linked [11], [15], [16].

**Figure 1:**
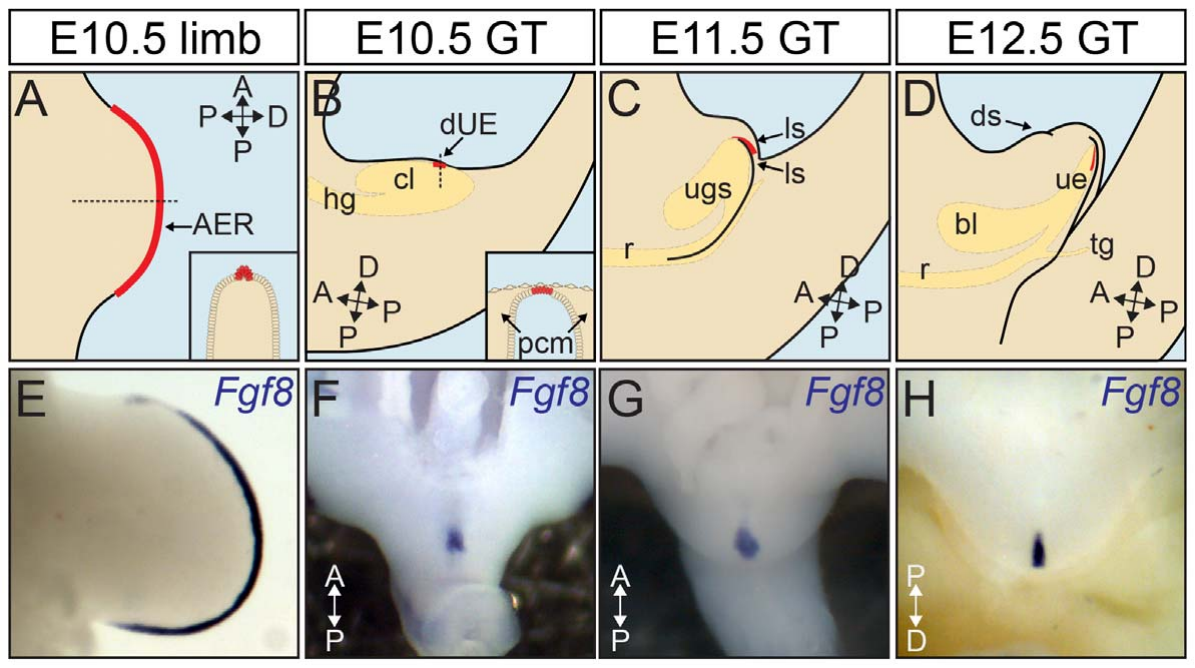
Distal signaling epithelia in the outgrowth of body appendages. (A) The outgrowth of the limb is instructed by a specialized ectodermal epithelium, the AER (in red), positioned at the distal edge of the limb bud expanding across the anteroposterior axis. Inset shows a cross section through the plain indicated by arrow. (B) The *Fgf8*-expressing GT signaling center dUE (in red) is positioned at the distal most part of the cloacal endoderm right below the ventral ectoderm (inset, coronal section through the plain indicated by arrow). (C, D) The dUE remains at the distal end of the urethral epithelium as the GT and the urethra undergo continuous proximodistal outgrowth. Endodermal cloaca was illustrated in yellow in B–D. (E–H) *Fgf8* expression marks the AER (E) and dUE (F–H). bl, bladder; ls, lateral swellings; ds, dorsal swelling; ugs, urogenital sinus; ue, urethral epithelium; r, rectum; hg, hindgut; tg, tail gut; PCM, para-cloacal mesenchyme.

The first step in appendage outgrowth is the establishment of an independent proximodistal developmental axis apart from the main body axis. This process requires precise regulation from instructive signals, which often come from a distal signaling center. In addition to promoting and maintaining outgrowth, these signals also provide directional information that determines the orientation and shape of future structures. Moreover, genes required for subsequent patterning and differentiation are often regulated by the distal signaling center. For example, during limb development, the initiation and continuous outgrowth of the limb bud rely on growth factors secreted from a strip of ectodermal epithelium, termed the apical ectodermal ridge (AER), positioned at the distal edge of limb bud (Figure 1A and 1E). Fibroblast Growth Factors (FGFs) are crucial signals provided by the AER, as FGF-soaked beads can replace the AER to induce limb outgrowth [17]–[20]. Furthermore, the AER FGF signals are obligatory to maintain a positive feedback loop involving SHH and Gremlin that coordinates patterning and growth of the limb [21]–[23].

The early GT is built by two mesenchymal swellings at either side of the cloacal membrane, which is later joined by a third outgrowth anterior to the cloacal membrane (Figure 1B–1D). Unlike limb, subsequent outgrowth of the GT has to accommodate a continuous extension of the cloacal endoderm, which forms the epithelial lining of the future urethral tube. This unique pattern of GT development suggests that the centrally located distal cloacal endoderm (later the distal urethral epithelium or dUE, marked in red in Figure 1B–1D, also shown by *Fgf8* in situ in Figure 1F–1H) is a strategic place for GT outgrowth. Consistently, an earlier study demonstrated that *Fgf8* is expressed in a strip of cells located at the distal-most part of the cloacal endoderm right below the ventral ectoderm (Figure 1B inset). Subsequent functional analyses revealed that physically removing *Fgf8*-expressing cells or application of neutralizing FGF8 antibody could abolish GT growth in organ culture, and this growth inhibition could be reversed by adding FGF8-soaked beads [24].

Along with these studies, we uncovered that activity of the canonical WNT-β-catenin pathway is restricted to the *Fgf8*-expressing distal cloacal endoderm and later the dUE [4]. We found that abolishing β-catenin (*β-Cat*) in the cloacal endoderm caused GT agenesis/reduction, whereas ectopic activation of the same pathway resulted in GT over-development. Interestingly, the site and level of WNT signaling activity positively correlated with *Fgf8* expression and the extent of outgrowth in both limbs and GT. These findings illustrated a parallel between dUE and AER signaling during appendage outgrowth. However, the exact mechanisms and functional relevance for this WNT-*Fgf8* regulation remain to be elucidated.

Recently, two independent studies reported a surprising observation that abolishing *Fgf8* [25], [26], or simultaneously abolishing *Fgf4* and *Fgf8* [26], in the cloacal endoderm does not affect GT outgrowth or GT-specific gene expression. These results questioned the relevance of FGF signals in external genitalia development and challenged the view that GT formation is organized and maintained by the dUE, which further suggested that the mechanisms underpinning limb and GT outgrowth are indeed divergent [2].

Herein, we adopted comprehensive genetic approaches to address the inductive signals in GT development, in comparison with that of the limb. We sought to define the role for FGF signaling and dUE-expressed *Fgf8* in the GT, and explore mechanisms upstream of FGF activation in the dUE of the GT and AER of the limb. Results from our analyses revealed a remarkably conserved *Wnt-Sp8-Fgf8* genetic circuitry that is crucial for proximodistal outgrowth of both paired- and unpaired-type appendages in mice.

## Results

### FGF Responsiveness Is Indispensable for GT Development

**Figure 2:**
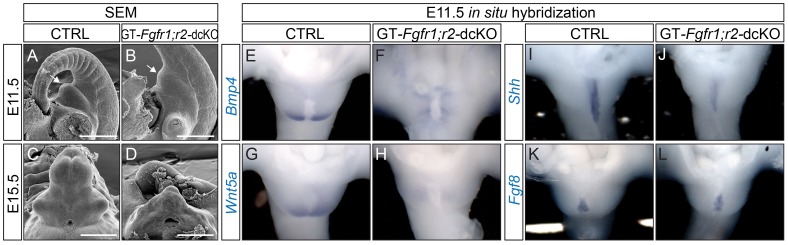
Defective GT development in GT-*Fgfr1*;*r2*-dcKO embryos. (A–F) SEM analyses on age-matched control (A, C) and dcKO mutants (B, D) showing a retarded proximodistal outgrowth in the mutants. (E–L) Whole mount in situ hybridization on E11.5 control and dcKO mutants using probes indicated. Note the downregulation of *Bmp4* and *Wnt5a* in the PCM (F and H), and *Shh* in the UE of the dcKO mutants (J). The dUE-*Fgf8* expression in the dcKO (L) was comparable to the control (K). Bars represent 500 µm.

Previous studies demonstrated that inactivating one or two FGF ligands did not affect genital development [25], [26]. We reasoned that the extensive genetic redundancy among FGF ligands might have undermined the power of these experiments. Therefore, we sought to re-evaluate the function of FGF signaling in GT development by conditionally abolishing FGF receptors. *Fgfr1* and *Fgfr2* are the only FGF receptors expressed in the developing GT [27]. Both *Fgfr1* and *Fgfr2IIIc* are expressed in the para-cloacal mesenchyme (PCM) during GT outgrowth [27], and *Fgfr2IIIb* is expressed in both the PCM and the urethral epithelium (UE) [27], [28]. To abolish all FGF responsiveness in the developing GT, we employed an *Msx2rtTA*;*tetO-Cre* system which enables doxycycline (Dox)-inducible gene ablation in both the PCM and the UE [29], [30]. Dox was given to pregnant females on embryonic day (E) 9.5 and E10.5 to induce Cre-mediated recombination of the *Fgfr1^f/f^* [31] and *Fgfr2^f/f^* [32] alleles. The phenotypes of *Msx2rtTA*;*tetO-Cre*;*Fgfr1^f/f^*;*Fgfr2^f/f^* mutant embryos [hereafter referred to as GT-*Fgfr1/2*-double conditional knockouts (cKO), or dcKO] were analyzed by scanning electron microscopy (SEM). GT-*Fgfr1*;*r2*-dcKO genital tubercles were underdeveloped compared to their littermate controls at all stages examined starting from E11.5, when a clear tubercle structure could first be detected (Figure 2B). At E12.5, the dcKO GTs showed a clear deficiency in proximodistal outgrowth, as they were much shorter than the controls (data not shown). At E15.5, the mutant GTs were smaller in size, deformed (Figure 2D) and lacked the characteristic mesenchymal patterning present in controls (Figure S1A–S1B). To further explore the cellular basis for the reduction in GT size, we analyzed proliferation and cell-death in the GT of these mutants. We performed phospho-histone H3 (PHH3) staining on E11.0 coronal GT sections and counted the number of PHH3+ cells in a fixed area. A 28% reduction in PHH3-positive cell number in the genital mesenchyme of the dcKOs was observed (Figure S1C-S1E, n≥10, p = 0.0017). We also performed TUNEL analyses but did not observe any differences in the number of apoptotic cells between control and dcKO mutant GTs (data not shown).

An examination of genes known to mediate genital tubercle initiation revealed alterations in normal gene expression patterns as early as E11.5. *Bmp4*, *Wnt5a* and *Msx1* were expressed in the PCM in control GTs (Figure 2E and 2G, and Figure S1F), and their expression was barely detectable in dcKO GTs (Figure 2F and 2H, and Figure S1G). *Msx2* was expressed in both the PCM and UE in controls (Figure S1H). In the GT-*Fgfr1*;*r2*-dcKOs, *Msx2* expression was absent in the PCM and downregulated in the UE (Figure S1I). Moreover, UE expression of *Shh* was also downregulated in the dcKOs (Figure 2I–2H). In contrast, dUE-*Fgf8* expression remained unchanged in the GT-*Fgfr1*;*r2*-dcKOs (Figure 2K–2L), suggesting that maintenance of *Fgf8* expression is independent of FGFR1 and FGFR2 during GT development. Collectively, these data demonstrate that FGF signaling is obligatory for promoting proximodistal GT outgrowth and for maintaining genital-specific gene expression.

### FGF Targets Genital Mesenchyme during GT Outgrowth

The *Msx2rtTA;tetO-Cre* system mediates gene deletion in both the cloacal endoderm as well as the PCM. Therefore, it is not clear whether changes observed in the aforementioned *Fgfr1;r2-*dcKOs were direct results of compromised FGF responsiveness in the PCM, or secondary to loss of FGF receptors in the cloacal endoderm. Thus, we tested the requirement for FGF responsiveness in these two compartments by using a previously characterized endoderm-specific *Shh^EGFPCre^* allele [4], [33], and a PCM-specific *Dermo1^Cre^* allele [4], [32], respectively. *Shh^EGFPCre/+^*;*Fgfr1^f/f^*;*Fgfr2^f/f^* (UE-*Fgfr1*;*r2*-dcKO) and *Dermo1^Cre/+^*;*Fgfr1^f/f^*;*Fgfr2^f/f^* (PCM-*Fgfr1*;*r2*-dcKO) mutants were generated and their GTs analyzed. Intriguingly, GTs from UE-*Fgfr1*;*r2*-dcKOs did not exhibit any morphological abnormalities in the early outgrowth phase, and their size and shape were comparable to stage-matched controls (Figure S2A–S2D). Histological analysis revealed normal patterning of the genital mesenchyme in UE-*Fgfr1*;*r2*-dcKOs embryos (Figure S2E and S2F). The only phenotype observed in these mutants was the abnormal maturation of urethral epithelium similar to what was observed in *Fgfr2IIIb* mutants [28], which will be discussed in a separate manuscript. Furthermore, regulatory genes including *Msx2*, *Bmp4*, *Wnt5a* and *Fgf8*, were also properly expressed in these UE-*Fgfr1*;*r2*-dcKO embryos (Figure S2G–S2J).

In contrast, the GTs of PCM-*Fgfr1*;*r2*-dcKO were clearly smaller than their littermate controls (Figure S3A–S3B). Further analyses revealed that the distal GT mesenchymal expression of P-ERK1/2, a previously established FGF target gene [26], was also downregulated in these mutants (Figure S3C–S3D). In addition, PHH3 staining on E11.5 embryos revealed a 20% reduction in mitotic figure number in the PCM-*Fgfr1*;*r2*-dcKOs (Figure S3E–S3G). Collectively, these data indicate that the main target for FGF signaling during GT outgrowth is the PCM. A similar requirement for FGF signaling in the limb mesenchyme has been described previously [34].

### FGF8 Is Sufficient to Regulate GT Development

**Figure 3:**
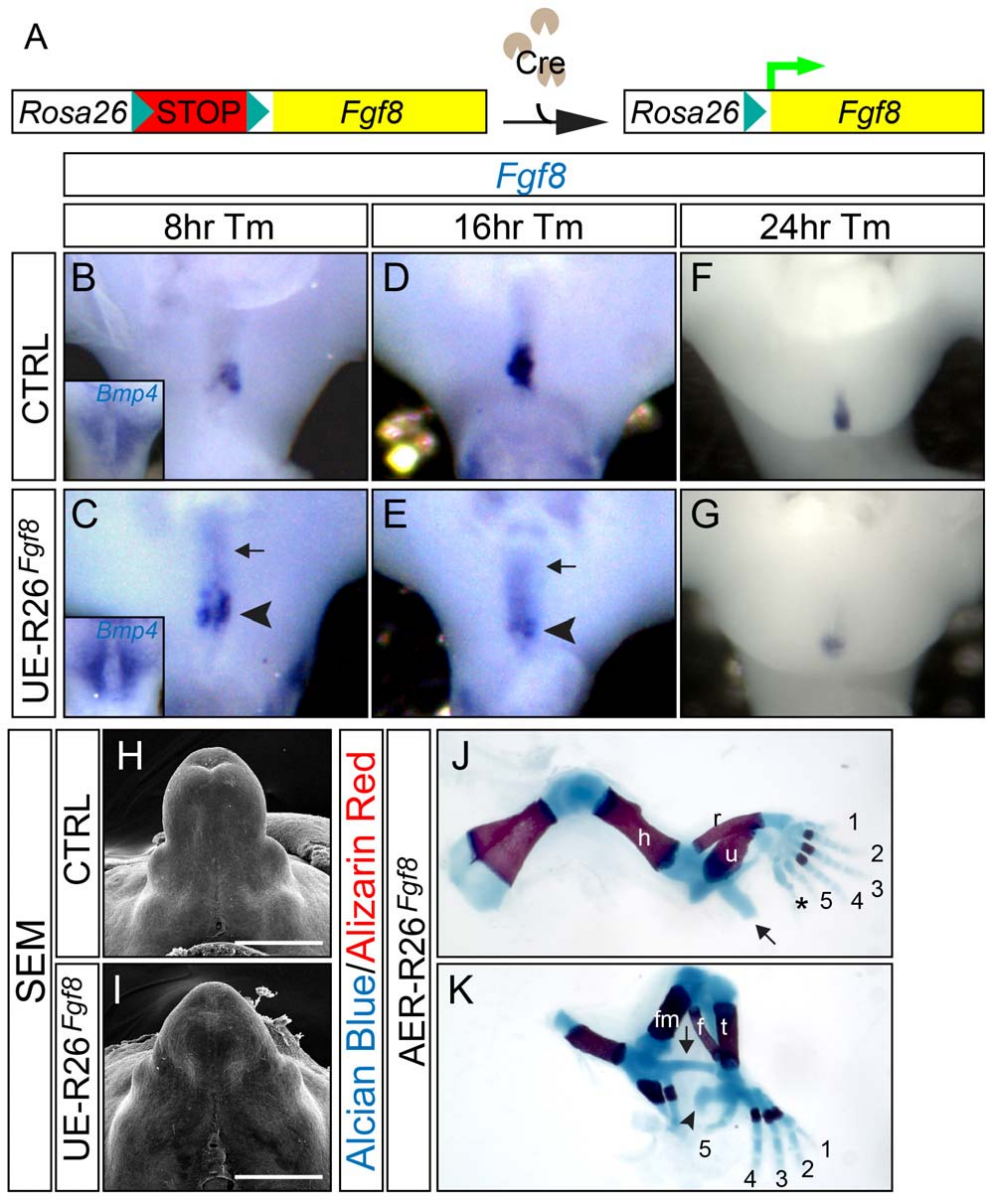
Appendage over-development in the *R26^Fgf8^*-GOF mutants. (A) A schematic diagram for *R26^Fgf8^* allele. (B–G) Whole mount in situ analyses on control (B, D, and F) and UE-*R26^Fgf8^* (C, E, and G) embryos at consecutive time points after Tm treatment. Note the upregulation of PCM-*Bmp4* ( inset in C) and dUE-*Fgf8* (C, arrowhead) in the UE-*R26^Fgf8^* embryos 8 hours after Tm administration, and a downregulation of dUE-*Fgf8* expression in the UE-*R26^Fgf8^* mutants 16 (E, arrowhead) and 24 hours (G) after Tm treatment. The ectopic *Fgf8* expression in the anterior cloacal endoderm is indicated by arrows in C and E. (H–I) SEM on E14.5 control (H) and UE-*R26^Fgf8^* GT (I). Note that the GT in the GOF mutant is bigger. (J–K) Skeleton staining of E18.5 AER-*R26^Fgf8^*-GOF embryos showing excessive limb development in the mutants. The postaxial extra digit in the forelimb is indicated by an asterisk in J, and the ectopic skeleton components were indicated by arrows in J and K. The arrowhead in K indicates an enlarged calcaneus. h, humerus; r, radius; u, ulna; fm, femur; t, tibia; f, fibula. Bars represent 500 µm.

*Fgf8* is normally expressed in the distal-posterior cloacal endoderm at E10.5, and then in the dUE through E11.5–E14.5. We sought to determine whether the PCM could respond to dUE-expressed *Fgf8* in vivo. We generated a conditional *Fgf8* overexpressor mouse line by knocking the *Fgf8* full-length cDNA (Accession: BC048734) into the ubiquitously expressed *Rosa26* locus, preceded by a floxed transcriptional stop cassette (*R26^Fgf8^*). This design allows ectopic *Fgf8* expression upon Cre-mediated recombination (Figure 3A). We used a tamoxifen (Tm)-inducible *Shh^CreERT2^* allele [4], [33] and generated *Shh^CreERT2^*/*^+^*;*R26^Fgf8^* gain of function (GOF) mutants (UE-*R26^Fgf8^*-GOF), to achieve UE-specific *Fgf8* overexpression upon Tm treatment at E10.5. The Cre expression domain of this *Shh^CreERT2^* allele recapitulates endogenous *Shh* expression, which includes all cloacal endodermal cells. Eight hours after Tm administration, we noted a clear upregulation (arrowhead in Figure 3C) and an anterior expansion (arrow in Figure 3C) of *Fgf8* expression in the cloacal endoderm evidenced by whole-mount in situ hybridization. The ectopic expression in the anterior cloacal endoderm (arrow in Figure 3C and 3E) was the result of ectopic *Fgf8* expression from the *R26^Fgf8^* allele. Notably, a concurrently augmented *Bmp4* expression was evident in the PCM (insets in Figure 3B and 3C). Sixteen hours after the initial tamoxifen injection, we observed a 19% increase in mitotic index in the PCM (Figure S4A–S4C, n = 10, p = 0.009). Consistently, the mutant GTs were larger than controls at E12.5 (Figure S4D–S4E) and E14.5 (Figure 3H–3I), respectively. These findings clearly demonstrate that endodermally expressed FGF8 can mediate gene expression and promote cell proliferation in the neighboring PCM.

Interestingly, we noted that endogenous *Fgf8* expression was differentially regulated in the UE-*R26^Fgf8^*-GOF mutants. Sixteen hours after Tm treatment, we observed a distinct downregulation of *Fgf8* expression in the GOF mutant dUE (arrowhead in Figure 3E) that persisted 24 (Figure 3G) and 48 hours after treatment (data not shown). It is also noteworthy that transcription from the *R26* locus was much weaker than that from the endogenous *Fgf8* locus, evidenced by the faint signal in the anterior cloacal endoderm (indicated by arrows in Figure 3C and 3E). Collectively, these findings demonstrate that the developing GT is sensitive to changes in FGF dosage, and a feedback loop is deployed to ensure proper signaling activity when misregulation occurs.

To compare the function of FGF8 in the limb and GT, we mated the *R26^Fgf8^* allele with a transgenic *Msx2-Cre* line [35], which confers *Cre* expression in the forming and mature AER and the ventral limb ectoderm. As expected, the AER-*R26^Fgf8^*-GOF mutants exhibited excessive limb growth and developed extra digits (asterisk in Figure 3J), an enlarged calcaneus bone (arrowhead in Figure 3K), and ectopic skeletal elements (arrows in Figure 3J–3K, and Figure S4G) in both forelimbs and hindlimbs. These overgrowth phenotypes are similar but more severe than what has been observed in the AER-*Fgf4*-GOF embryos [36], and further support the concept that FGF8 plays a pivotal role in promoting the outgrowth of both appendages.

### FGF8 Is the Main Signal Output for the Canonical WNT Pathway in the Distal Signaling Epithelia of the Appendages

**Figure 4:**
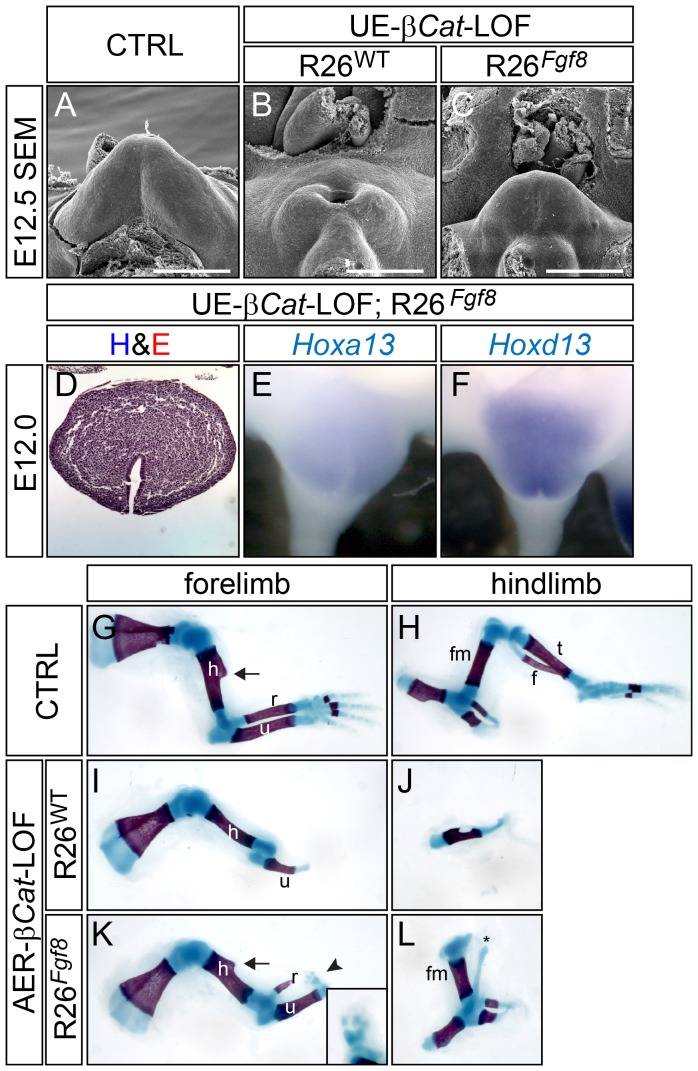
FGF8 rescues appendage reduction in β-catenin-LOF mutants, but not in *Shh*-KO mutants. (A–C) SEM analyses revealed an absence of GT in UE-β*-Cat*-LOFs (B), and a distinct tubercle structure in the LOF mutant carrying *R26^Fgf8^* allele (C). (D–F) Histological and in situ analyses on E12.0 *β-Cat*-LOF;*R26^Fgf8^* GT showing a normal patterning (D) and *Hox* genes expression (E and F). (G–L) Skeleton preparation on E18.5 embryos showing an absence of autopod and radius, truncation of ulna, underdeveloped humerus in the forelimb (I), and a complete absence of all stylopod, zeugopod and autopod elements in the hindlimb of AER*-β-Cat*-LOF mutant (J); and a presence of autopod rudiments (arrow and inset in K) and radius with proper humerus and ulna in the forelimb (K), and a fully developed femur in the hindlimb of *R26^Fgf8^*-rescued *β-Cat*-LOF mutant (L). h, humerus; r, radius; u, ulna; fm, femur; t, tibia; f, fibula. Bars represent 500 µm.

Our previous work has shown that the WNT-β-catenin signaling pathway and *Fgf8* expression are tightly coupled in the distal signaling centers both in the limb and in the GT [4]. The above finding that *Fgf8* was repressed by its own overexpression was in sharp contrast to our previous observation in the UE-*β-Cat*-GOF mutants where ectopic up-regulation *Fgf8* was sustained in the UE [4]. This suggested that the canonical WNT pathway plays a key role in controlling the *Fgf8* auto-regulatory feedback loop. Together, these observations suggest that the WNT-*Fgf8* regulatory relationship is essential for appendage formation, and prompted us to test whether loss of *Fgf8* was the critical event causing appendage reduction in embryos deficient in *β-Cat* in the AER and the UE. Therefore, we attempted to restore *Fgf8* expression in *β-Cat* loss of function (LOF) embryos and analyze its effect on appendage formation. We generated *Shh^EGFPCre/+^*; *β-Cat^f/f^*;*R26^Fgf8/+^* (UE-*β-Cat*-LOF;*R26^Fgf8^*) mutants and analyzed their GT development. In contrast to absence of the GT in the UE-*β-Cat*-LOF embryos (Figure 4B), a cone-shaped tubercle structure was readily discernible in compound mutants carrying the *R26^Fgf8^* allele (Figure 4A–4C). To determine whether this rescued structure bears GT characteristics, we performed both histological and gene expression analyses. Hematoxylin and Eosin (H&E) stained E12.0 transverse GT sections showed that the morphology of the rescued GT closely resembled that of the controls with the urethra properly positioned at the ventral side of the GT (Figure 4D). In addition, *Hoxa13* and *Hoxd13*, both markers of the GT, were expressed in the rescued genital tubercles (Figure 4E and 4F). Altogether, these data indicate that restoring FGF8 rescued GT agenesis caused by β-catenin deficiency. It is noteworthy that this rescue of *β-Cat*-cKO by FGF8 is confined to the GT, as other caudal malformations observed in the *β-Cat*-cKO including failed cloaca septation and defective tail formation, were still present in the UE-*β-Cat*-LOF;*R26^Fgf8^* embryos (data not shown).

To test whether forced *Fgf8* expression can also rescue limb deficiency caused by *β-Cat* ablation, we generated *Msx2-Cre*; *β-Cat^f/f^* (AER*-β-Cat*-LOF) and *Msx2-Cre*; *β-Cat^f/f^*;*R26^Fgf8/+^* (AER*-β-Cat*-LOF;*R26^Fgf8^*) mutants. Consistent with a previous investigation [3], all autopod elements, radius, and distal two-thirds of the ulna were missing from the forelimbs of AER-*β-Cat*-LOFs (Figure 4I), whereas the humerus was thinner and lacked the deltoid tuberosity (Figure 4I compared to G, arrow). In contrast, the LOF embryos with the *R26^Fgf8^* allele developed normal humeri with the deltoid tuberosity (Figure 4K, arrow). The radius was evident and the ulna was longer and thicker (Figure 4K). Moreover, several small pieces of alcian blue-stained cartilage were observed distal to the ulna, indicating the presence of autopod rudiments (arrowhead and inset in Figure 4K). The hindlimbs of the AER-*β-Cat*-LOFs were largely absent except for a small remnant of the pelvic girdle (Figure 4J), whereas the *R26^Fgf8^*-expressing *β-Cat*-LOF embryos developed near normal pelvic girdles and femurs (Figure 4L) along with one or two ectopic cartilages (asterisk in Figure 4L). These phenotypes were consistently observed in all AER*-β-Cat*-LOF;*R26^Fgf8^* embryos (n = 10), and indicated that exogenously supplying FGF8 can partially restore distal limb structures lost in the AER-*β-Cat*-LOF embryos. Collectively, these data suggest that FGF8 can promote outgrowth of both the GT and the limb in the absence of canonical epithelial WNT activity, suggesting that it functions as a downstream effector of WNT signaling during limb and GT outgrowth.

### Growth-Promoting Role for SHH Is Independent of FGF during Appendage Outgrowth

The positive feedback loop involving FGF*s* and SHH plays a critical role in appendage outgrowth, as evidenced by the down-regulation in *Fgf* expression in both the AER [21], [22], [35], [37] and the dUE [5], [6] of *Shh*-KOs, which display reductions in both appendages. We next examined whether forced *Fgf8* expression could also rescue the severe appendage deficiencies caused by the absence of SHH. We generated *Shh^CreERT2/CreERT2^*;*R26^Fgf8/+^* (*Shh*-KO;UE-*R26^Fgf8^*) embryos, which allowed us to induce *Fgf8* expression in the UE of *Shh* null mutants (*Shh^CreERT2^* allele is also a null allele). However, we detected neither tubercle formation (Figure S5B), nor *Hoxa13* or *Hoxd13* expression (Figure S5C and S5D) in the cloacal region of these compound mutants at E12.5, 48 hours after Tm treatment. We also generated *Msx2-Cre*; *R26^Fgf8/+^*; *Shh^CreERT2/CreERT2^* (*Shh*-KO;AER-*R26^Fgf8^*) mutants, to test whether sustaining *Fgf8* expression in the AER of *Shh* mutants can restore limb development. We carefully analyzed four *Shh*-KO embryos carrying both *Msx2-Cre* and *R26^Fgf8^* alleles, and found no evidence of more advanced limb development (Figure S5G and S5H), compared to thirteen *Shh*-KO mutants without *R26^Fgf8^* alleles (Figure S5E and S5F).Together, these results indicated that although *Shh* and *Fgf8* expression are interdependent during appendage outgrowth, their function in promoting appendage outgrowth is independent and non-redundant.

### GT-Specific Gene Expression Depends on dUE-FGF Signals

**Figure 5:**
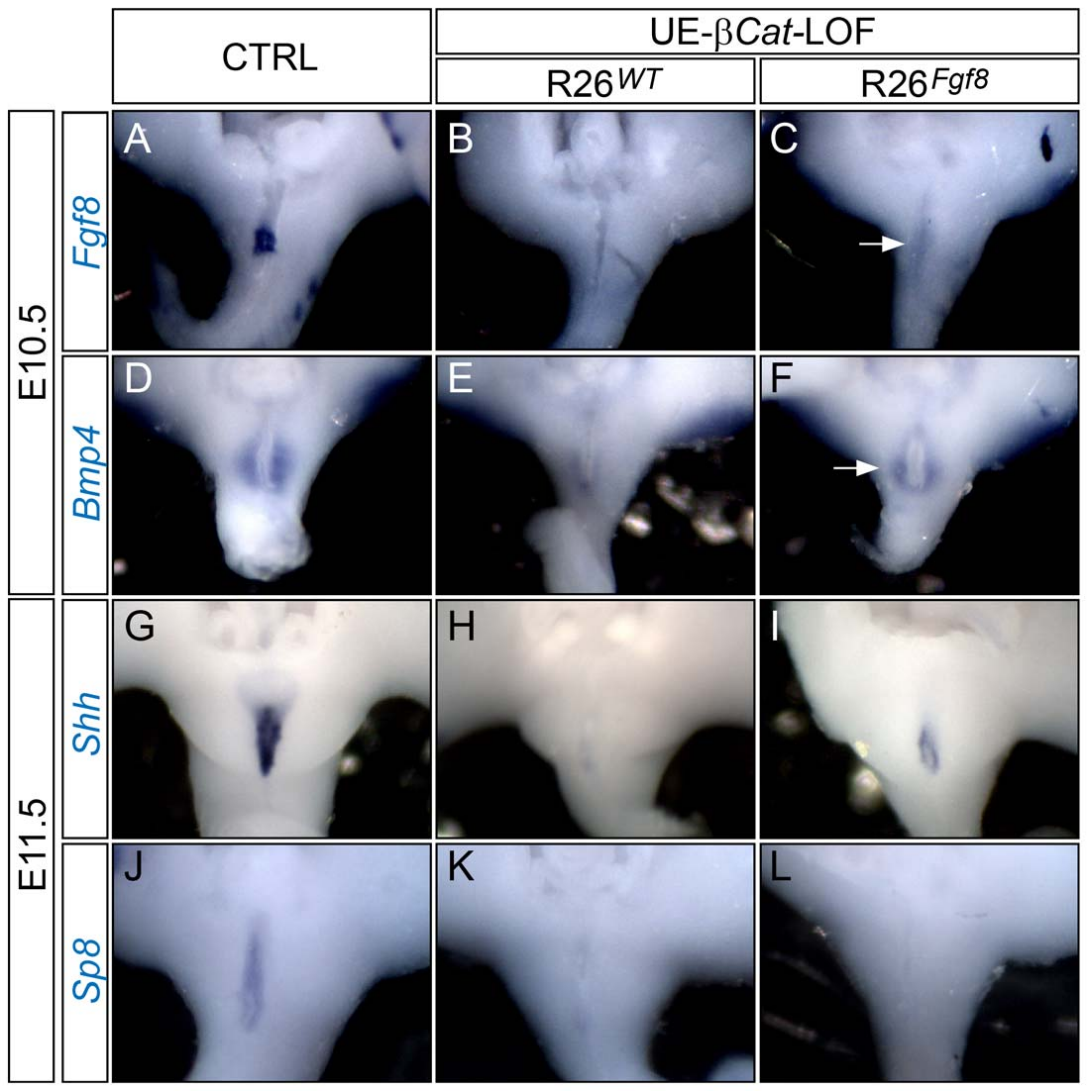
GT gene expression analyses on UE*-β-Cat-*LOF embryos with forced *Fgf8* expression. (A–L) Whole mount in situ analyses using probes indicated. Note that the LOF mutants lack cloacal endoderm/UE *Fgf8* (B) and *Shh* (H) expression, and PCM *Bmp4* (E) expression; whereas weak *Fgf8* expression (C), partially restored PCM *Bmp4* (F) expression, and UE *Shh* (I) expression can be detected in the LOF mutants with *R26^Fgf8^* allele. Also note the lack of *Sp8* expression in LOF mutants with (L) or without (K) *R26^Fgf8^* allele.

We next analyzed GT gene expression in UE-*β-Cat*-LOF; *R26^Fgf8^* mutants to further interrogate the genetic networks underlying GT development and identify downstream targets of dUE signaling. *Fgf8* expression was detected in the distal cloacal endoderm in controls (Figure 5A), but was absent in UE-*β-Cat*-LOF mutant (Figure 5B) at E10.5. The *R26^Fgf8^*-expressing LOF embryos, on the other hand, showed very weak *Fgf8* expression throughout the cloacal endoderm (arrow in Figure 5C). This low-expression was consistent with what we observed in the UE- *R26^Fgf8^*-GOF embryos, suggesting that these *Fgf8* transcripts were transcribed from the *R26* locus. Expression of *Bmp4* and *Wnt5a* was normally detected in the PCM (Figure 5D and Figure S6A), absent in the UE-*β-Cat*-LOFs (Figure 5E and Figure S6B), and partially restored by ectopically supplying *Fgf8* expression from the *R26^Fgf8^* allele (Figure 5F and Figure S6C). The SHH pathway was also compromised in the *β-Cat*-LOF mutants. *Shh* (Figure 5H) and *Ptch1* expressions (Figure S6E) were markedly decreased in the cloacal endoderm and the PCM, respectively. On the other hand, in the LOF embryos with the *R26^Fgf8^* allele, the expression of *Shh* was partially restored in the UE (Figure 5I) and the PCM expressed *Ptch1* restored to a level comparable to controls (Figure 5F).

Combined, these data indicate that most genes differentially regulated in the UE-*β-Cat*-LOF mutants were responsive to FGF8 induction, suggesting that their expression is controlled by the dUE-FGF signals. However, we found that *Sp8*, a transcription factor normally expressed in the cloacal endoderm (Figure 5J), was lost in the UE of UE-*β-Cat*-LOF mutant (Figure 5K), and did not respond to FGF8 supplementation (Figure 5L).

### SP8 Is Required for Appendage Outgrowth

**Figure 6:**
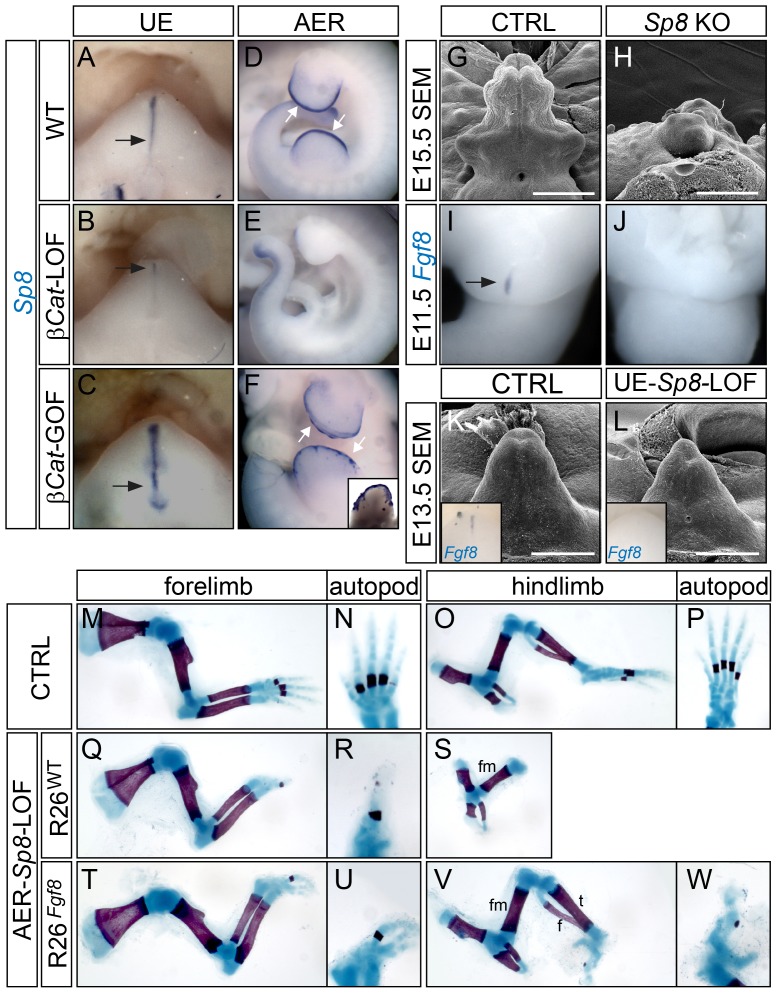
Appendage deficiencies in *Sp8* mutants. (A–F) Whole mount *Sp8* in situ showing a downregulation of *Sp8* expression in the UE and the AER of corresponding *β-Cat*-LOF mutants (B, E), and an upregulation of *Sp8* expression in the UE (C) and the ventral limb ectoderm (F) of *β-Cat*-GOF mutants. Note the tail expression of *Sp8* remains unchanged in (E compared to D). (G, H) SEM on E15.5 embryos showing an absence of GT in the *Sp8* KO mutant (H). (I, J) *Fgf8* in situ showing loss of expression in E11.0 *Sp8* KO mutant. (K, L) SEM on E13.5 embryos showing a mild distal reduction in the UE-*Sp8*-LOF (L). Insets showing whole mount *Fgf8* in situ on E12.5 control (K) and UE-*Sp8*-LOF (L) GTs. Note the downregulation of *Fgf8* in the UE-*Sp8*-LOF mutant (L). (M–W) Skeletal preparation of E18.5 embryos showing that AER-*Sp8*-LOF lacked most autopod elements in the forelimb (Q and R), and all zeugopod and autopod elements in the hindlimb (S); whereas in the *R26^Fgf8^*-rescued LOF mutants, several digits were formed in the forelimb (T, U), and the tibia and fibula were fully developed along with some autopod rudiments in the hindlimb (V, W). h, humerus; r, radius; u, ulna; fm, femur; t, tibia; f, fibula. Bars represent 500 µm in G and H, 400 µm in K and L.

*Sp8* is expressed throughout the cloacal endoderm and later in the UE (Figure 6A), overlapping with the *Fgf8*-expressing dUE during GT development. Previous studies have implicated SP8 in the transcriptional regulation of *Fgf8* expression in the mouse commissural plate [38] and the chick limb ectoderm [39]. These findings prompted us to explore its role in mediating the WNT-*Fgf8* pathway. We first examined *Sp8* expression in the UE-*β-Cat*-LOF and GOF-mutants (for GOF analyses, we used a previously established *β-Cat^Δex3^* allele which produces stabilized β-catenin upon Cre-mediated recombination [40]). We found that *Sp8* expression was reduced in the *β-Cat*-LOF UE (Figure 6B) and increased in the *β-Cat^Δex3^* GOF UE (Figure 6C). Similarly, robust *Sp8* expression was also detected in the AER (Figure 6D), markedly reduced in the AER- *β-Cat*-LOF mutants (Figure 6E), and upregulated in the *β-Cat^Δex3^* GOF mutants where β-catenin activity was ectopically augmented in the limb ectoderm (Figure 6F, inset showing ventral view of a hindlimb). These data indicated that *Sp8* is downstream of Wnt-β-catenin signaling in the UE and the limb ectoderm.

Next, we examined the GT phenotype of *Sp8*-null (KO) mutants. We found that 14/36 mutant embryos examined between E12.5–E15.5 exhibited GT agenesis (Figure 6H), while the rest demonstrated a range of GT defects including deformation, hypoplasia and proximal hypospadias (data not shown). *Fgf8* expression was completely absent in the cloacal endoderm in all *Sp8* KOs examined at E11.5 (Figure 6J, n = 9). Notably, these embryos also exhibited other caudal malformations such as deformed perineum, anal channel and tails, raising the concern whether the observed GT defects were secondary to earlier cloacal or neural tube malformations. Thus, we employed a conditional *Sp8* null allele with the *Shh^CreERT2^* line to generate UE-*Sp8*-LOF mutants, and induced *Sp8* deletion by Tm treatment around the time of GT initiation (E10.5). GTs from the UE-*Sp8*-LOF mutants were smaller than their age-matched controls, especially at the distal tip (Figure 6L). We also found a clear reduction in *Fgf8* expression in these mutants by in situ analyses (inset in Figure 6L). Consistently, the expression domains of *Wnt5a* (Figure S7B) and *Msx2* (Figure S7D), both maintained by FGF8 from the neighboring dUE, were reduced.

Limb truncation, attributed to a failure to form the AER and consequently loss of *Fgf8* expression, has been previously described in *Sp8−/−* embryos [41]. We generated AER-*Sp8*-LOFs using the *Msx2-Cre* line, and these mutants also showed a defect in limb outgrowth as evidenced by a loss of distal structures (Figure 6Q–6S). The stylopod and zeugopod developed normally in the forelimbs, while typically only one abnormal digit formed in these mutants (Figure 6Q–6R). The tibia and fibula were either missing or severely truncated, and no autopod was observed in the hindlimbs (Figure 6S). Notably, these limb defects can also be partially rescued by over expression of *Fgf8*. Skeleton preparation of E18.5 embryos showed that the AER-*Sp8*-LOF embryos carrying *R26^Fgf8^* allele developed three digits in the forelimb (Figure 6U), and full-length tibia and fibula along with several irregular autopod elements in the hindlimbs (Figure 6V–6W). Altogether, these data indicated that *Sp8* is required to maintain *Fgf8* expression and appendage outgrowth in the distal signaling center of both the limb and GT.

### *Sp8* Mediates WNT-Induced *Fgf8* Expression

**Figure 7:**
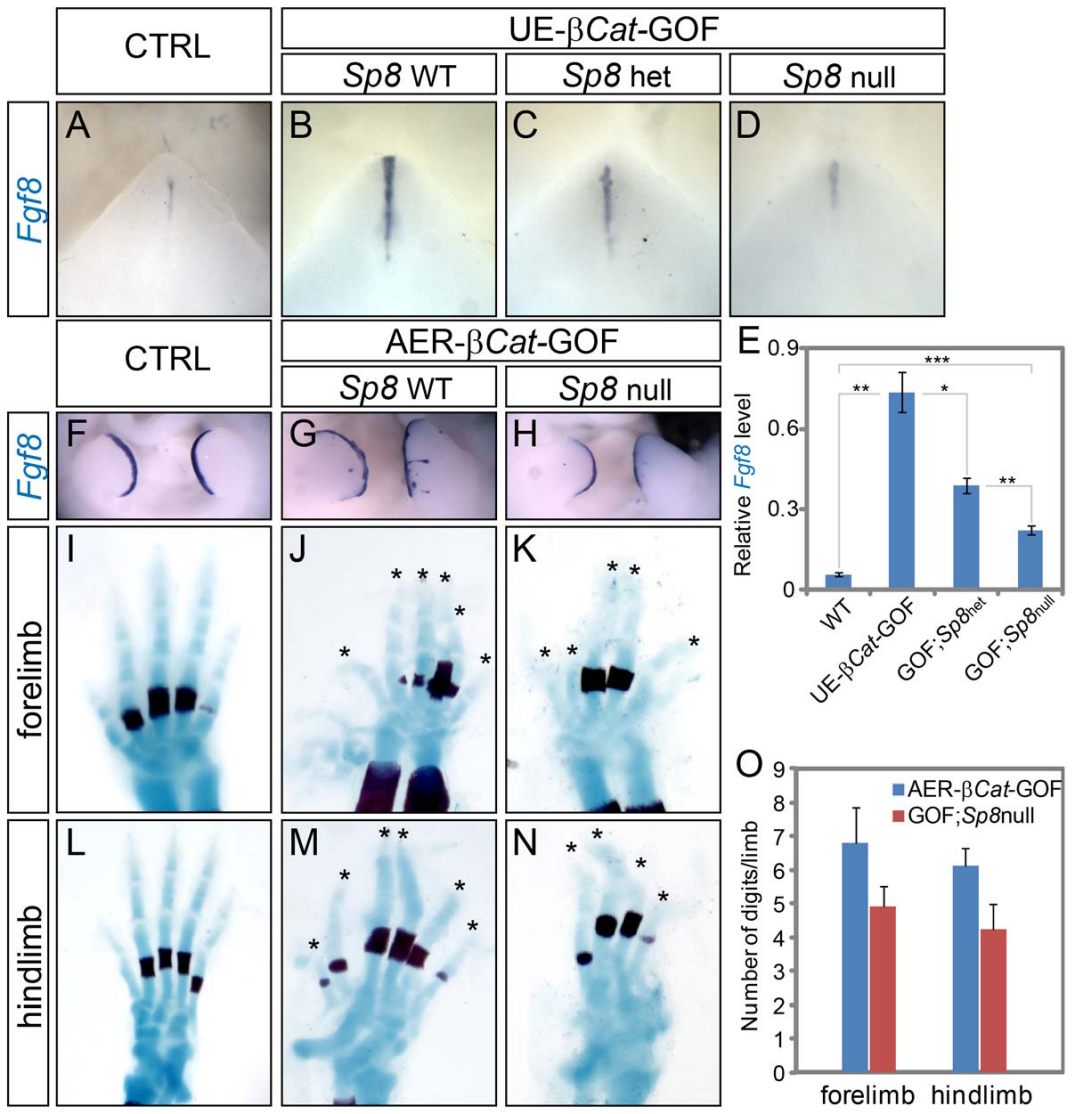
WNT-induced ectopic *Fgf8* expression was attenuated in *Sp8* conditional knockouts. (A–D) Whole mount *Fgf8* expression on E12.5 GTs from embryos with genotypes indicated. Note the gradual reduction in *Fgf8* expression in correlation with *Sp8* allele number from B to D. (E) Real time-RT PCR analyses on E12.5 GTs with genotypes indicated. (F–H) Whole mount *Fgf8* in situ on E11.5 limbs showing expanded expression in the AER-*β-Cat*-GOF mutant (G), and near normal expression in the GOF embryos with both *Sp8* alleles abolished (H). (I–N) The autopod phenotype of AER*-β-Cat*-GOF mutants with (J, M) or without *Sp8* (K, N) alleles. Quantification of digit numbers is shown in O.

To test whether *Sp8* is in the same genetic pathway with β-catenin and *Fgf8*, we sought to determine whether SP8 mediates WNT-induced *Fgf8* expression in the dUE and AER. We generated compound mutants using the *β-Cat^Δex3^* allele, which we have previously shown to activate *Fgf8* expression in both the UE and the limb ectoderm, together with the floxed *Sp8* allele and the corresponding Cre lines. We compared *Fgf8* expression in embryos with one *β-Cat^Δex3^* allele and different numbers of functional *Sp8* alleles by real-time RT-PCR and in situ hybridization. We found that the level of WNT-induced *Fgf8* expression in the UE positively correlated with the number of functioning *Sp8* alleles (Figure 7A–7E). Removing one wild type *Sp8* allele caused a twofold reduction in *Fgf8* expression, whereas ablating both wild type *Sp8* alleles reduced *Fgf8* expression by more than three-fold (Figure 7E). These results were further verified by *Fgf8* in situ hybridization (Figure 7A–7D).

Similarly, deleting both *Sp8* alleles abolished the ectopic *Fgf8* expansion in the limb ectoderm of *Msx2-Cre*; *β-Cat^Δex3/+^* (AER-*β-Cat*-GOF) embryos (Figure 7F–7H), and consequently attenuated the polysyndactyly phenotype caused by constitutively active canonical WNT signaling (Figure 7I–7O). With two wild type *Sp8* alleles, AER-*β-Cat*-GOF mutants developed an average of 6.8 digits in the forelimbs, and 6.1 digits in the hindlimbs (Figure 7J and 7M). In thirty percent of the embryos, ectopic limb in the flank ectoderm and ventral ectoderm were detected (Figure S8A and S8B). In comparison, AER-*β-Cat*-GOF;*Sp8*-null mutants only developed 4.9 digits in the forelimbs and 4.2 digits in the hindlimbs (Figure 7K and 7N, 28% and 31% reduction compared to AER-*β-Cat* GOFs, respectively). In addition, no extra limbs were observed at ectopic locations. Collectively, these results indicate that SP8 is responsible, at least in part, for the WNT-induced activation of *Fgf8* expression during appendage outgrowth.

**Figure 8:**
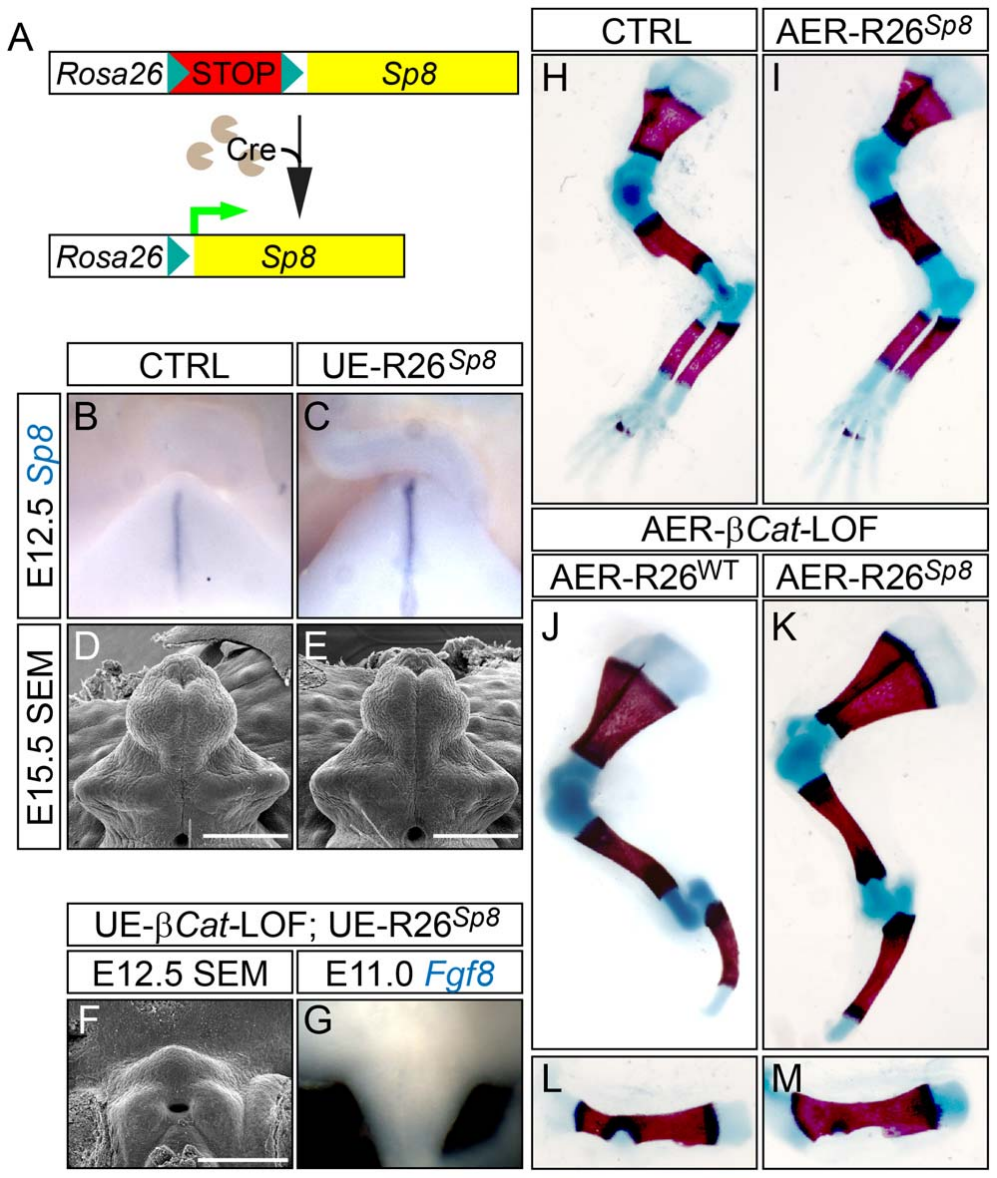
Construction and characterization of *R26^Sp8^* knock-in allele. (A) A schematic diagram for *R26^Sp8^* allele. (B, C) *Sp8* in situ on E12.5 GTs showing an upregulation of *Sp8* expression in the UE-*R26^Sp8^* embryos (C). (D–F) SEM analysis on E15.5 embryos showing comparable GT development between control and the UE-*R26^Sp8^* mutant (E), and no tubercle formation in the UE-*β-Cat*-LOF mutant carrying *R26^Sp8^* allele (F). (G) *Fgf8* in situ showing no *Fgf8* expression in the UE-*β-Cat*-LOF;*R26^Sp8^* mutant. (H–M) Skeletal preparation showing no difference between control (H) and AER-*R26^Sp8^* GOF mutant (I), or between the AER-*β-Cat*-LOF mutants with (K, M) or without the *R26^Sp8^* allele (J, L). Bars represent 500 µm in D and E, 400 µm in F.

To test whether augmented *Sp8* expression alone can induce *Fgf8* overexpression, we generated a *R26^Sp8^* allele to conditionally overexpress *Sp8* using the same strategy as for the *R26^Fgf8^* line (Figure 8A). Mice carrying *Shh^EGFPCre^* or *Msx2-Cre* alleles were used to generate corresponding UE-*R26^Sp8^*-GOF and AER-*R26^Sp8^*-GOF embryos. Overexpression of *Sp8* in the UE and AER was confirmed by in situ hybridization (Figure 8C, and data not shown). However, unlike Fgf8- or *β-Cat*-GOF embryos, *Sp8*-GOF embryos showed normal development in both appendages (Figure 8E and 8I). *Fgf8* expression in the dUE and the AER of the corresponding Sp8-GOF mutant was also comparable to their wild type counterparts (Figure S9A–S9D).

We also crossed the *R26^Sp8^* allele into the UE- or AER-*β-Cat*-LOF mutants to test whether forced *Sp8* expression can bypass epithelial β-catenin to induce *Fgf8* expression and initiate/maintain appendage outgrowth. We carefully analyzed six compound mutants and did not observe phenotypic rescue in either the GT (Figure 8F) or the limb (Figure 8J–8M). In addition, no *Fgf8* induction was detected in the dUE of the corresponding *β-Cat*-LOF mutants carrying *R26^Sp8^* allele (Figure 8G). All of these results indicate that SP8 by itself is insufficient to activate *Fgf8* expression, and suggest that SP8 is a facilitator of WNT-mediated *Fgf8* activation during appendage formation.

## Discussion

In this study, we investigated the molecular cascade that regulates distal signaling centers in appendage development. Our study elicited a conservative genetic circuitry involving WNT-β-catenin signaling, the transcription factor *Sp8*, and the growth factor *Fgf8*, that underpins proximodistal outgrowth of limbs and external genitalia.

### FGF8 Mediates FGF Signaling in GT Outgrowth

Our work provides in vivo evidence that FGF signaling is indispensible for early GT outgrowth. These findings are consistent with results from organ culture studies that inhibition of FGF signaling caused an arrest in GT development [24]. Our data demonstrates that the PCM's ability to respond to an FGF signal is essential for normal early genital tubercle outgrowth, as removing FGF receptors from the PCM caused impaired cell proliferation and perturbed normal gene expression patterns which led to severe GT reduction.

The obligatory role for FGF signaling during tubercle morphogenesis underscores the importance of identifying FGF ligands important for GT development. Our data are consistent with the views of Haraguchi et. al. [24], suggesting that dUE-expressed *Fgf8* plays a key role in promoting GT outgrowth. *Fgf8* is expressed at the correct time and place to signal to the PCM, which expresses both *Fgfr1* and *Fgfr2*. This is in conflict with recent data by Seifert et. al. [25] in which they suggested that a normal GT could develop in the absence of *Fgf8* expression. They did not detect FGF8 protein, which led to the conclusion that the *Fgf8* mRNA may be present but not actively translated by the UE. They proposed that the ventral ectoderm may be an alternative source of other FGF ligands. In contrast, our results indicate that FGF8 protein can be made by the cloacal endoderm/UE as we demonstrated that a weakly-expressed *R26^Fgf8^* allele can profoundly alter cell proliferation and gene expression in the neighboring PCM, and rescue GT agenesis in β-catenin mutants. In addition, our observation that the endogenous *Fgf8* promoter is repressed upon forced *Fgf8* overexpression revealed that the developing GT is equipped with mechanisms that can fine-tune the level of FGF8 signaling. All of these data indicate that the GT is not only responsive but also sensitive to FGF8 during normal development. The inability to detect FGF8 in the dUE by IHC is likely caused by the low expression level of *Fgf8*. We found that in E10.5 mouse embryos, robust AER *Fgf8*-expression was observed 2 hours after incubation with Alkaline Phosphatase substrates following standard in situ hybridization procedures, whereas dUE-expression was not observed until 12–18 hours later. In addition, the extensive genetic redundancy among FGFs has to be carefully considered. A recent study demonstrated ectopic *Fgf4* expression in the dUE of *Fgf8*-cKO embryos, and ectopic *Fgf3* expression in the dUE of *Fgf4;Fgf8*-dcKO embryos [26]. Both FGF4 and FGF3 can efficiently induce mitogenic activity when paired with FGFR1 or FGFR2 in vitro [42]. These observations indicate that not only FGFs endogenous to the dUE can compensate for the loss of *Fgf8*, other FGFs can also be ectopically activated to fulfill the requirement for FGF signaling. Activities from these ectopic FGFs could well explain the lack of GT phenotype in *Fgf8*-cKO mutants. Intriguingly, induction of *Fgf3* and *Fgf4* was not observed in either *β-Cat*- or *Sp8*-cKOs (Figure S10A–S10D), while the dUE expression of *Fgf9* was downregulated in *Sp8*-cKOs (Figure S10E–S10F). These results suggest that the upregulation of these compensatory FGF factors also requires WNT and SP8. Alternatively, the hypothesis that FGFs can be produced by the ventral ectoderm is plausible [25]. However, one has to keep in mind that the GT is built and patterned around the UE. Most regulatory genes expressed distally including *Msx1*, *Msx2*, *Wnt5a* and *Lef1*, showed peri-dUE expression but not sub-ectodermal expression. Consistently, we have shown that the expression of these genes is orchestrated by UE-specific WNT and FGF signaling. Considering all the available evidence, we conclude that FGF8 produced by the dUE is most likely the endogenous ligand that mediates FGF responses during GT development.

Notably, the GT phenotype of the UE-*Fgfr1;r2*-dcKO embryos is less severe than what was observed previously in *Fgfr2IIIb*-KOs [28]. This difference is likely attributable to the method of gene ablation since in the *Fgfr2IIIb*-KO embryos, *Fgfr2-IIIb* is abolished from not only the UE, but also from the ventral ectoderm. The underdeveloped phenotype described in *Fgfr2IIIb*-KO embryos reflects a deficiency from ectodermal FGF responsiveness as it can be phenocopied by conditional ablation of both *Fgfr1* and *Fgfr2* using the ectodermally restricted *Msx2-Cre* allele (Lin et. al., unpublished data).

### Implication of a Conserved WNT-SP8-FGF8 Pathway in Appendage Outgrowth

We have demonstrated a conserved WNT-SP8-FGF8 pathway in the distal signaling epithelia that functions to promote proximodistal outgrowth of both limbs and genitalia. We and others have shown that the canonical WNT pathway is a master molecular switch in the signaling epithelia during appendage formation, as epithelial WNT activation is not only necessary but also sufficient to induce *Sp8* and *Fgf8* expression and appendage outgrowth. FGF8 is the downstream signal output for the WNT pathway during this process as it acts directly on recipient mesenchymal cells to promote cell proliferation and establish patterns of gene expression. This genetic hierarchy has been supported by our observation that forced overexpression of *Fgf8*, even at a level much lower than endogenous expression, can bypass the requirement for epithelial WNT-β-catenin signaling to activate gene expression and initiate/maintain appendage outgrowth. Notably, in both appendages, the rescue of β-catenin deficiency by ectopic *Fgf8* expression is only partial. This could be due to either weak *Fgf8* expression from the *R26* locus compared to the endogenous level of expression, or the possibility that in addition to regulating *Fgf8* expression, canonical WNT signaling is also required for the formation of the AER structure, independent of FGF signaling [20]. The regulation of *Fgf8* by the canonical WNT-β-catenin signaling pathway is in part mediated by SP8, as *Sp8* expression is regulated by WNT signaling in both limb ectoderm and the UE, and is necessary for *Fgf8* expression and subsequent appendage formation. However, unlike WNT activity and *Fgf8* expression, the expression domain of *Sp8* is not restricted to the AER and dUE but also includes the limb ectoderm [41] as well as proximal UE (Figure 6A), suggesting that SP8 is a permissive but not inductive factor for the establishment of *Fgf8* expression. In support of this notion, overexpressing *Sp8* in the limb ectoderm or UE did not cause any perturbation in *Fgf8* expression or appendage formation. In addition, forced *Sp8* expression failed to rescue *Fgf8* expression and appendage outgrowth in the epithelial *β-Cat*-LOF mutants.

Notably, even with both *Sp8* alleles mutated, the dUE and AER *Fgf8* expression in the *β-Cat*-GOF mutants is still higher than in the controls. This finding apparently is counterintuitive to the obligatory role for SP8 in maintaining *Fgf8* expression during normal outgrowth processes. One possible explanation is that the level of WNT signaling induced by the stabilized β-catenin protein from the *β-Cat^Δex3^* allele might be too high, and not subjected to endogenous regulatory mechanisms. This could potentially trigger ectopic events that lead to *Fgf8* overexpression. It is also noteworthy that the other members of SP/KLF transcription factor family *Sp6*, was upregulated in the *β-Cat*-GOF mutants (Figure S11). *Sp6* is expressed in the cloacal endoderm and the AER in early appendage development (Figure S11B and Figure S6A, respectively), and loss of *Sp6* causes abnormal AER formation and *Fgf8* expression [43]. Similar to *Sp8*, the expression of *Sp6* is also downstream of the canonical WNT signaling pathway but independent of FGFs in the AER [43]. The exact function of SP6 in regulating GT development and dUE-*Fgf8* expression remains to be determined. However, it is plausible that high levels of SP6 induced by ectopic WNT signaling might compensate for the absence of SP8 in the regulation of *Fgf8* expression.

The exact molecular mechanisms through which *Fgf8* expression is regulated by SP8 and β-catenin, and how *Sp8* expression is regulated by WNT-β-catenin remain to be determined. Direct binding of the β-catenin/LEF1 complex to cis-regulatory elements within the *Fgf8* promoter has been reported in dental epithelial cell lines [44] and nephron progenitors [45], suggesting that this WNT-*Fgf8* pathway also functions in the development of other organs involving epithelial-mesenchymal interactions. In our preliminary studies, we identified several novel LEF binding sites around *Fgf8* and *Sp8* genes (data not shown). The functional relevance of these binding sites in GT and limb development will be the goal of future investigations.

Although the distal signaling cascades appear to be similar in both appendages, the upstream events leading to their initiation are likely different. In the limb, the ectoderm is the site for both *Wnt3* expression [3], [46], and the induction of canonical Wnt downstream targets as evidenced by TOPGAL activity [47]. On the other hand, the establishment of the dUE signaling center in the cloacal endoderm appears to involve signal transduction between the cloacal endoderm and the ventral ectoderm. Both *TOPGAL* and *Fgf8* expression are confined to the endodermal cells adjacent to the genital ectoderm [4], [24], and the contact between ectoderm and endoderm appears to be a prerequisite for *Fgf8* induction and GT initiation [25]. In support of this notion, candidate WNT ligands responsible for activating canonical WNT signaling in the dUE, *Wnt3* and *Wnt7a*, are both expressed in the genital ectoderm [4], [26].

Altogether, our results demonstrate extensive parallels between genetic networks regulating the outgrowth of both the limb and GT. These findings strongly support the notion initially brought up by two pivotal studies on the role of Hox genes in appendage development [10], [11], that mammalian GT development appears to be achieved through co-option of the limb outgrowth program, i.e. the mammalian GT and limb share deep homology [48], [49].

## Materials and Methods

### Construction of *R26^Fgf8^* and *R26^Sp8^* Alleles

Clones containing full length *Fgf8* (BC048734) and *Sp8* (BC082582) cDNAs were obtained from Invitrogen (Carlsbad, CA). The cDNAs were released and subcloned into the *NotI* site of the pBigT vector [50]. The insert containing either *Fgf8* or *Sp8* cDNA and a floxed transcription stop cassette was released by *PacI/AscI* double digestion and cloned into pRosa26-PA [51]. The targeting construct was linearized by SwaI and subjected to electroporation (performed by ES cell core at Washington University). The ES cells were screened for recombination by PCR and southern blotting. Five out of seventy-two clones were positive for recombination for *R26^Fgf8^* allele, and six out of sixty clones were positive for *R26^Sp8^* allele. Positive clones were expanded, karyotyped and used for blastocyst injection. For both lines, at least three chimeras were able to pass the knock-in alleles through germline transmission, and the phenotypes resulting from expression of the knock-in alleles were identical. For all experiments described in the manuscript, three embryos with the same genotype were examined if not otherwise specified.

### Animal Maintenance and Treatments

All animals were maintained according to NIH guidelines and in compliance with animal protocol approved by Washington University. *Msx2rtTA* [29], *Sp8^f/f^* and *Sp8−/−* [41], *tetO-Cre* [52], *β-Cat^f/f^* [53], *β-Cat^ex3/ex3^* [40], *Shh^CreERT2^* and *Shh^EGFPCre^* [33], *Msx2-Cre* [35], *Fgfr1^f/f^* [31], *Dermo1^Cre^* and *Fgfr2^f/f^* [32] alleles were previously described. Tamoxifen treatment and doxycycline treatment were performed as previously described [30].

### Real-time RT–PCR

For all experiments, we used three independent biological samples from each genotype. Each sample contained a pool of RNA isolated from two E12.5 GTs with the corresponding genotype. The results were analyzed using the delta-CT method. Expression of the corresponding genes was normalized to that of housekeeping gene *Rpl7*.

### Whole-Mount In Situ Hybridization

Whole mount in situ hybridization was performed using a standard protocol. The probes were previously described [4].

### Anatomical and Histological Analyses

Paraformaldehyde (4%)-fixed embryos were dehydrated and embedded in paraffin. Five-micron transverse GT sections were generated using a standard microtome. Scanning electron microscopy analyses were performed as previously described [4].

### Skeleton Preparation

Skeleton preparation was performed as previously described [54].

### Accession Numbers

The Genbank accession numbers for genes included in the manuscript are as follows: *Fgf8* (NM_010205.2), *Sp8* (NM_177082.4), β-catenin (NM_007614.3), *Fgfr1* (NM_010206.2), *Fgfr2* (NM_010207.2), *Shh* (NM_009170.3).

## Supporting Information

Figure S1 Defective mesenchymal patterning and proliferation in GT-*Fgfr1;r2*-dcKO embryos. (A–B) E15.5 histological analyses showing patterned mesenchymal condensation in control GT (E), but not the dcKO GT (B). (C–E) PHH3 staining of E11.0 control and dcKO GTs showing a 28% reduction in the number of PHH3-positive mesenchymal cells in a fixed region (n≥10, p = 0.0017). (F–I) Whole mount in situ on E11.5 control and dcKOs showing downregulation of both *Msx1* and *Msx2* in the PCM of the mutants.
(JPG)

Figure S2 Phenotype of UE-*Fgfr1;r2*-dcKOs. (A–D) SEM analyses revealed no gross morphological differences between controls and the dcKO mutants at E13.5 (A, B), and E15.5 (C, D). (E, F) Histological analyses on E15.5 GT transverse sections showing no difference in size or patterning of the GT mesenchyme between the control (E) and mutant (F). (G–J) Whole mount in situ hybridization on E11.5 UE-*Fgfr1;r2*-dcKOs revealing normal expression of regulatory genes.
(JPG)

Figure S3 Defective GT outgrowth in the PCM-specific Dermo1-Cre; *Fgfr1;r2*-dcKOs. (A–B) SEM on E14.5 control and *PCM-Fgfr1;r2*-dcKO showing an underdeveloped GT in the mutant (B). Note that the P-D outgrowth was deficient in the mutant. (C–D) Phospho-ERK1/2 staining on E11.5 coronal GT sections showing downregulation of the P-ERK1/2 in the distal GT mesenchyme in the mutant (D). (E–G) PHH3 staining showed a 20% reduction in mitotic figure number in the dcKO mutants (p = 0.017).
(JPG)

Figure S4 Phenotype of AER- and UE- specific Fgf8-GOF mutants. (A–C) PHH3 staining on E11.0 control and UE-*R26^Fgf8/+^* embryos showing a 19% increase in number of PHH3-positive cells in the GOF GT(n = 10, p = 0.009). (D–E) SEM analyses on E12.5 UE-*R26^Fgf8/+^* and control GT showing an overdeveloped GT in the GOF mutant. (F–G) Skeletal preparation of E18.5 control and AER-*R26^Fgf8/+^* embryos showing ectopic bone (arrow) development.
(JPG)

Figure S5 Appendage phenotype of *Shh-KO* mutants with forced *Fgf8* expression. (A, B) SEM analyses showing no tubercle formation in *Shh*-KO mutants with (A) or without (B) *R26^Fgf8^* allele. (C, D) *Hoxa13* (C) and *Hoxd13* (D) in situ showing no expression in the *Shh*-KO bearing *R26^Fgf8^* allele. (E–H) Skeleton staining showing no difference in limb development between *Shh*-KOs with (E, F) or without (G, H) AER-*R26^Fgf8^* expression.
(JPG)

Figure S6 GT gene expression analyses on UE*-β-Cat-*LOF embryos with forced *Fgf8* expression. (A–F) Whole mount in situ using probes indicated. Note the absence of PCM *Wnt5a* (B) and *Ptch1* (E) expression in the LOF mutants; and partially restored *Wnt5a* (C) and near normal *Ptch1* (F) expression in the LOF mutants with *R26^Fgf8^* allele.
(JPG)

Figure S7 GT gene expression analyses on UE*-Sp8-*LOF embryos. (A–D) Whole mount in situ on E12.5 embryos using pobes indicated. Note the distal expression domains of both *Msx2* (B) and *Wnt5a* (D) were both smaller in the mutant GTs.
(JPG)

Figure S8 Excessive limb development in ectopic positions in the AER-*β-Cat*-GOF embryos. (A, B) Ectopic limb formation was indicated by arrows.
(JPG)

Figure S9 *Fgf8* expression in R26*^Sp8^*-GOF mutant. (A–D) *Fgf8* in situ on control (A, C), UE- R26*^Sp8^*-GOF (B) and AER-R26*^Sp8^*-GOF mutant (D) revealing comparable expression levels in the GT (A, B) and the forelimbs (C, D).
(JPG)

Figure S10 Expression of Fgfs in UE-*β-Cat*- and *Sp8*-cKOs (A–F) In situ hybridization using probes indicated. Note no *Fgf4* (A–B) or *Fgf3* (C–D) expression was detected in either mutants (A positive control for the *Fgf4* in situ is shown in the inset of A), and downregulation of dUE *Fgf9* expression (arrow in E) in *Sp8*-cKO (F).
(JPG)

Figure S11 *Sp6* expression in E11.0 WT embryos. (A, B) Whole mount *Sp6* in situ showing AER expression (arrows in A) and UE expression (B).
(JPG)
