# Supplementary figures and images for: Delineating a Conserved Genetic Cassette Promoting Outgrowth of Body Appendages
Source: PLoS Genet. 2013 Jan 24;9(1):e1003231. doi: 10.1371/journal.pgen.1003231 (PMC3554569; doi:10.1371/journal.pgen.1003231)

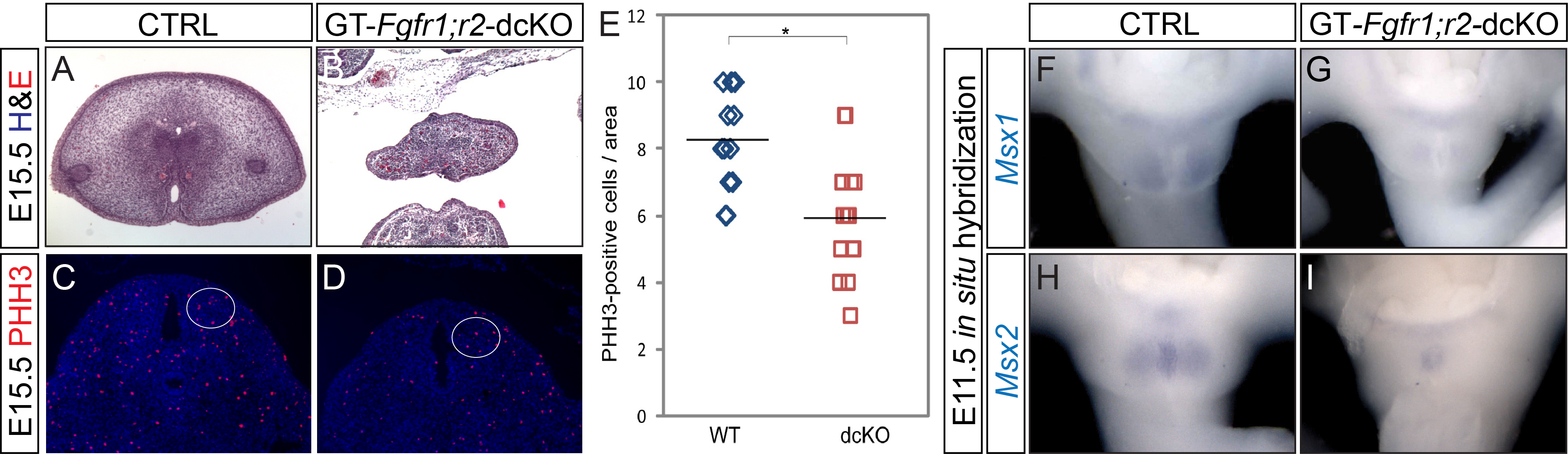

Supplement: Figure S1 — Defective mesenchymal patterning and proliferation in GT-Fgfr1;r2-dcKO embryos. (A–B) E15.5 histological analyses showing patterned mesenchymal condensation in control GT (E), but not the dcKO GT (B). (C–E) PHH3 staining of E11.0 control and dcKO GTs showing a 28% reduction in the number of PHH3-positive mesenchymal cells in a fixed region (n≥10, p = 0.0017). (F–I) Whole mount in situ on E11.5 control and dcKOs showing downregulation of both Msx1 and Msx2 in the PCM of the mutants. (JPG) [file pgen.1003231.s001.jpg]

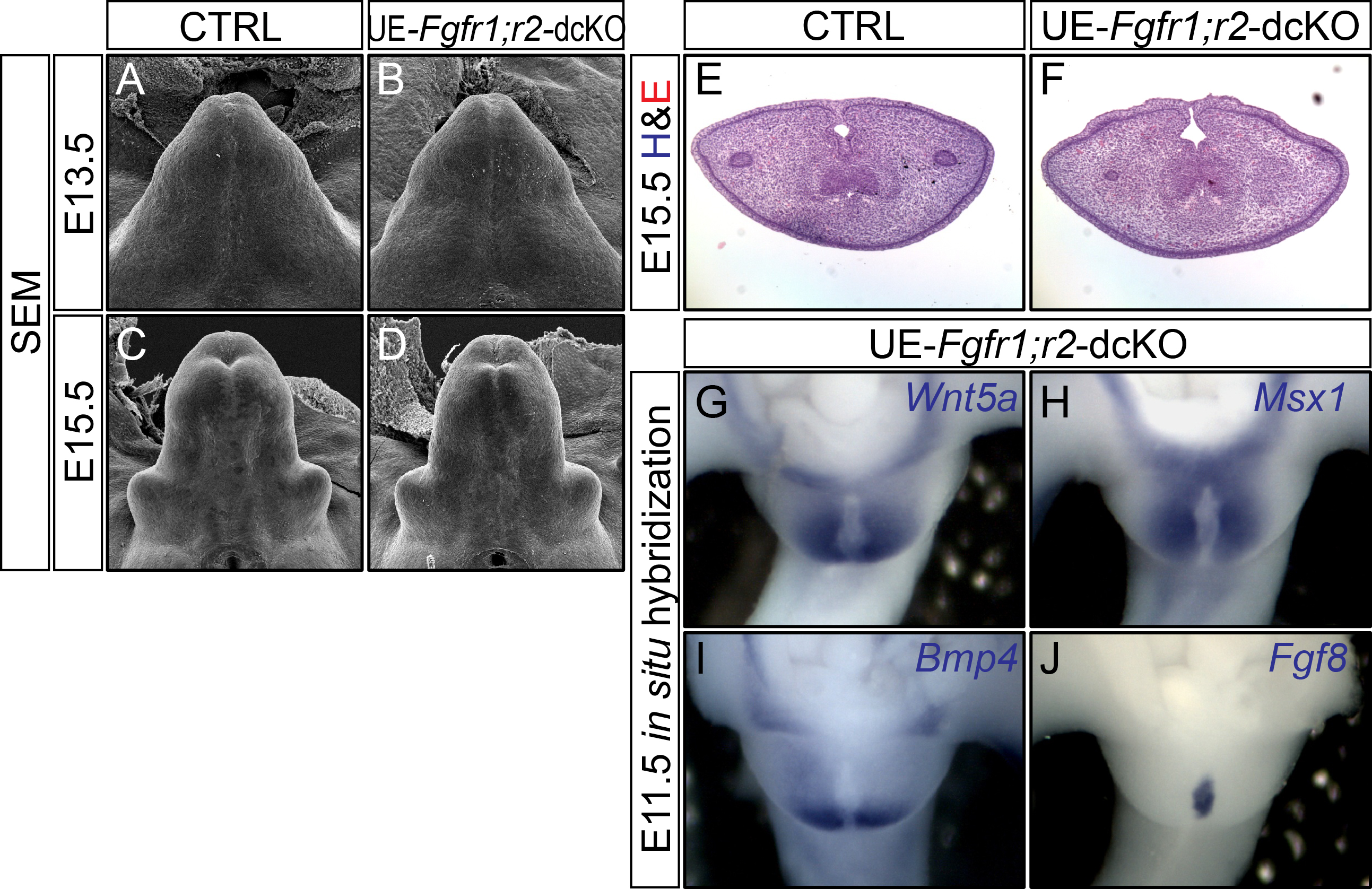

Supplement: Figure S2 — Phenotype of UE-Fgfr1;r2-dcKOs. (A–D) SEM analyses revealed no gross morphological differences between controls and the dcKO mutants at E13.5 (A, B), and E15.5 (C, D). (E, F) Histological analyses on E15.5 GT transverse sections showing no difference in size or patterning of the GT mesenchyme between the control (E) and mutant (F). (G–J) Whole mount in situ hybridization on E11.5 UE-Fgfr1;r2-dcKOs revealing normal expression of regulatory genes. (JPG) [file pgen.1003231.s002.jpg]

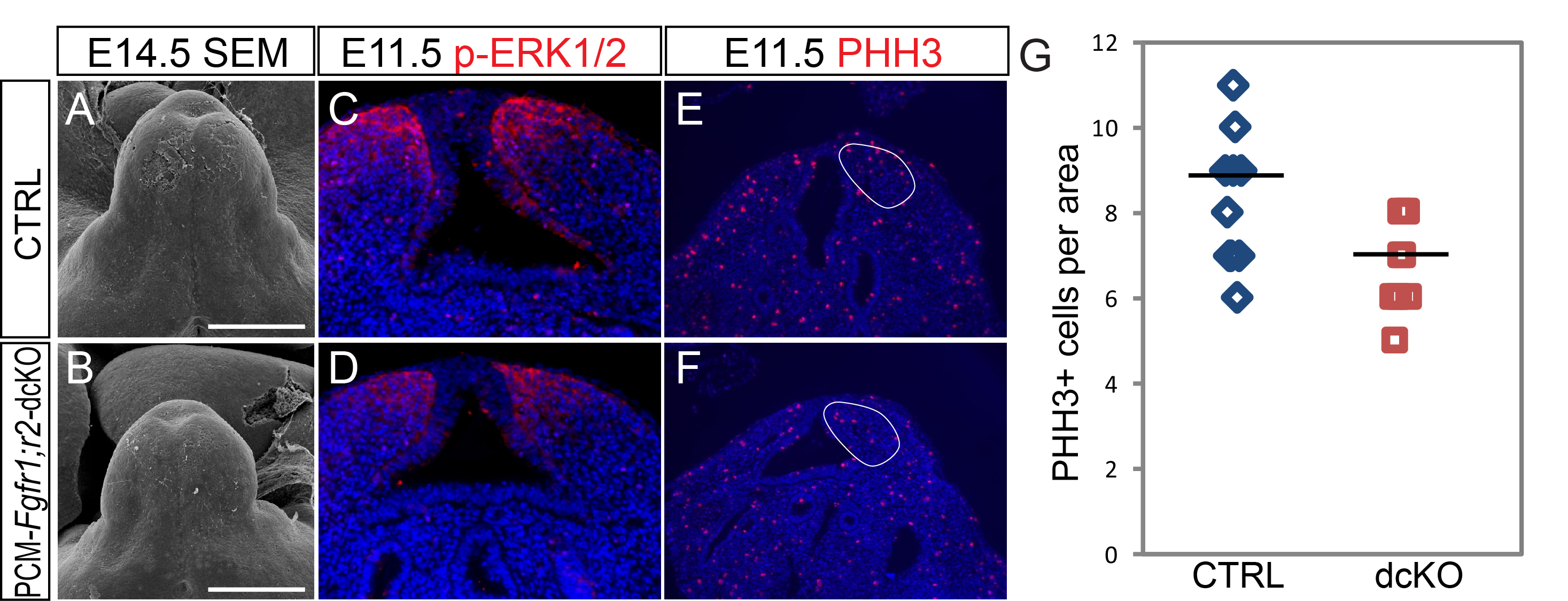

Supplement: Figure S3 — Defective GT outgrowth in the PCM-specific Dermo1-Cre; Fgfr1;r2-dcKOs. (A–B) SEM on E14.5 control and PCM-Fgfr1;r2-dcKO showing an underdeveloped GT in the mutant (B). Note that the P-D outgrowth was deficient in the mutant. (C–D) Phospho-ERK1/2 staining on E11.5 coronal GT sections showing downregulation of the P-ERK1/2 in the distal GT mesenchyme in the mutant (D). (E–G) PHH3 staining showed a 20% reduction in mitotic figure number in the dcKO mutants (p = 0.017). (JPG) [file pgen.1003231.s003.jpg]

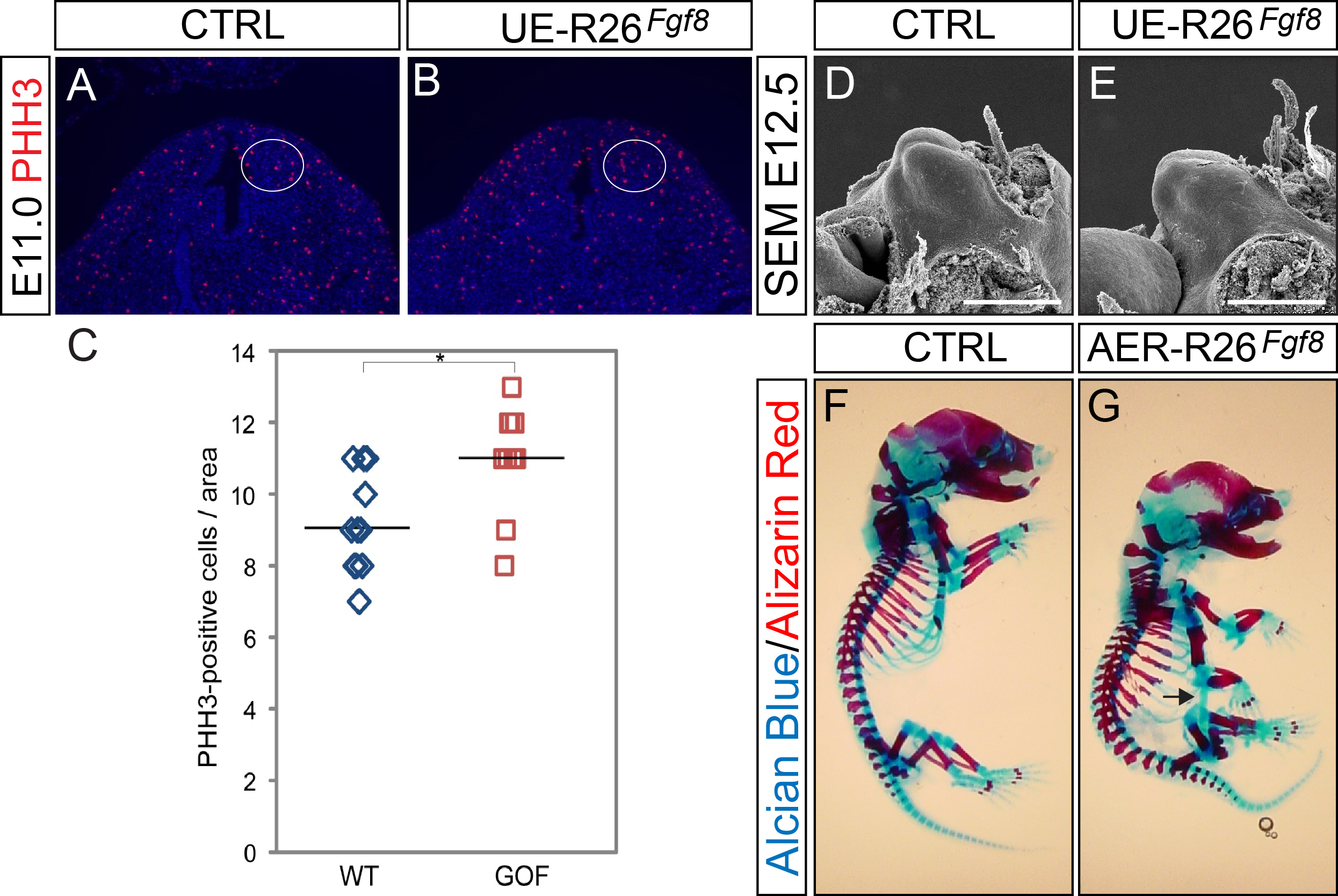

Supplement: Figure S4 — Phenotype of AER- and UE- specific Fgf8-GOF mutants. (A–C) PHH3 staining on E11.0 control and UE-R26Fgf8/+ embryos showing a 19% increase in number of PHH3-positive cells in the GOF GT(n = 10, p = 0.009). (D–E) SEM analyses on E12.5 UE-R26Fgf8/+ and control GT showing an overdeveloped GT in the GOF mutant. (F–G) Skeletal preparation of E18.5 control and AER-R26Fgf8/+ embryos showing ectopic bone (arrow) development. (JPG) [file pgen.1003231.s004.jpg]

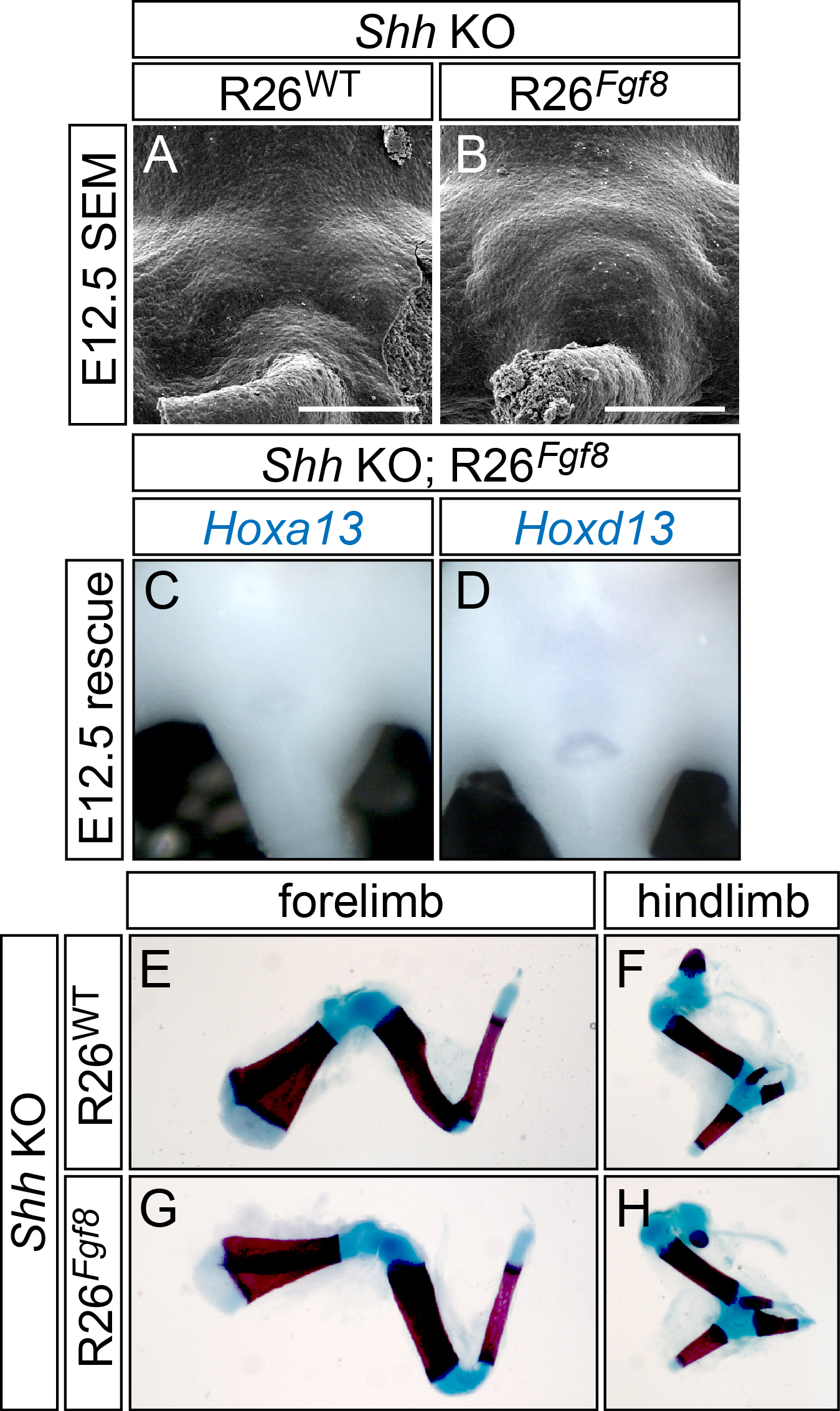

Supplement: Figure S5 — Appendage phenotype of Shh-KO mutants with forced Fgf8 expression. (A, B) SEM analyses showing no tubercle formation in Shh-KO mutants with (A) or without (B) R26Fgf8 allele. (C, D) Hoxa13 (C) and Hoxd13 (D) in situ showing no expression in the Shh-KO bearing R26Fgf8 allele. (E–H) Skeleton staining showing no difference in limb development between Shh-KOs with (E, F) or without (G, H) AER-R26Fgf8 expression. (JPG) [file pgen.1003231.s005.jpg]

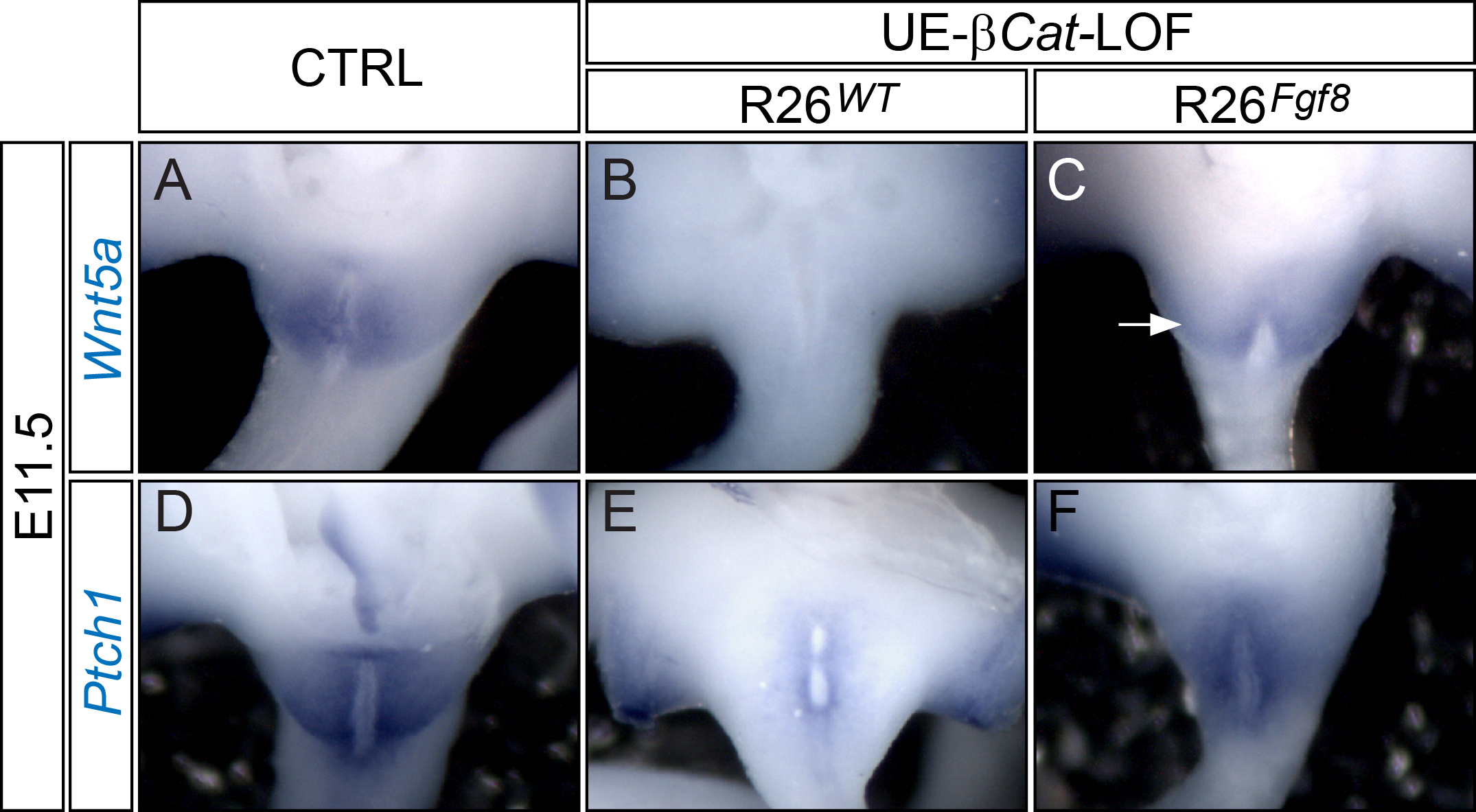

Supplement: Figure S6 — GT gene expression analyses on UE-β-Cat-LOF embryos with forced Fgf8 expression. (A–F) Whole mount in situ using probes indicated. Note the absence of PCM Wnt5a (B) and Ptch1 (E) expression in the LOF mutants; and partially restored Wnt5a (C) and near normal Ptch1 (F) expression in the LOF mutants with R26Fgf8 allele. (JPG) [file pgen.1003231.s006.jpg]

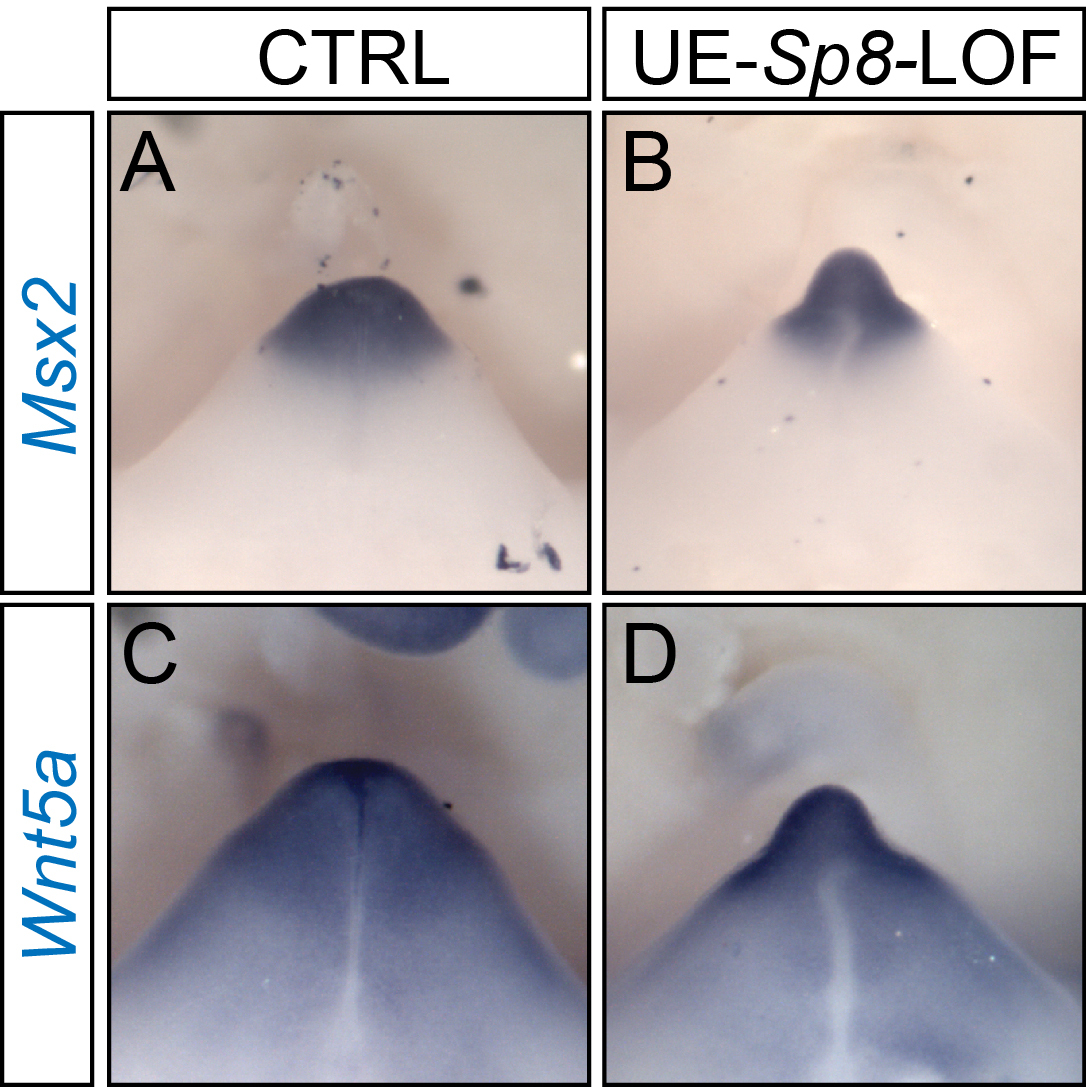

Supplement: Figure S7 — GT gene expression analyses on UE-Sp8-LOF embryos. (A–D) Whole mount in situ on E12.5 embryos using pobes indicated. Note the distal expression domains of both Msx2 (B) and Wnt5a (D) were both smaller in the mutant GTs. (JPG) [file pgen.1003231.s007.jpg]

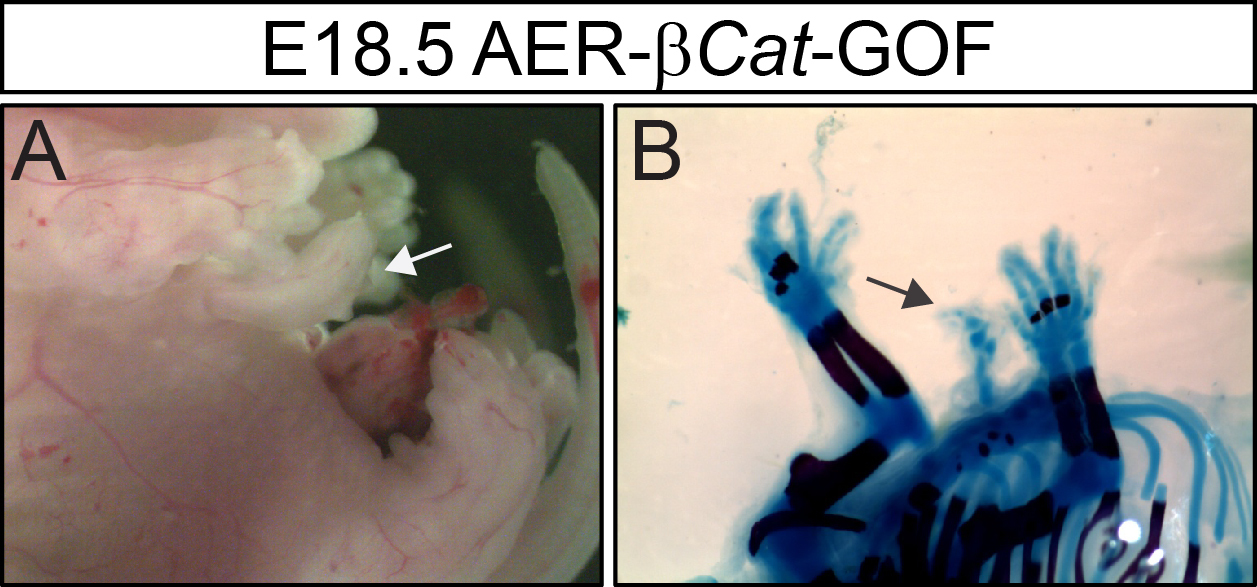

Supplement: Figure S8 — Excessive limb development in ectopic positions in the AER-β-Cat-GOF embryos. (A, B) Ectopic limb formation was indicated by arrows. (JPG) [file pgen.1003231.s008.jpg]

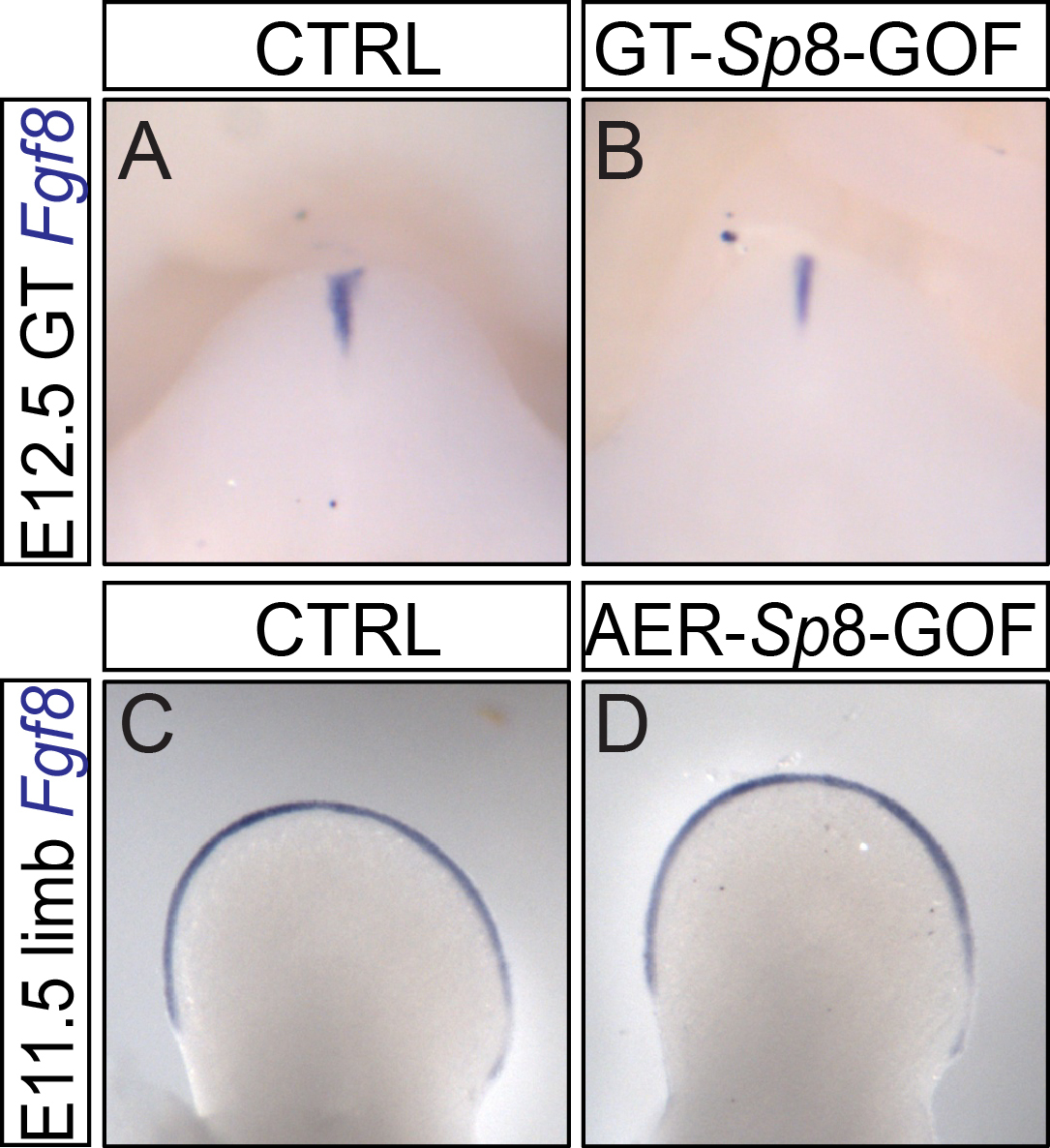

Supplement: Figure S9 — Fgf8 expression in R26Sp8-GOF mutant. (A–D) Fgf8 in situ on control (A, C), UE- R26Sp8-GOF (B) and AER-R26Sp8-GOF mutant (D) revealing comparable expression levels in the GT (A, B) and the forelimbs (C, D). (JPG) [file pgen.1003231.s009.jpg]

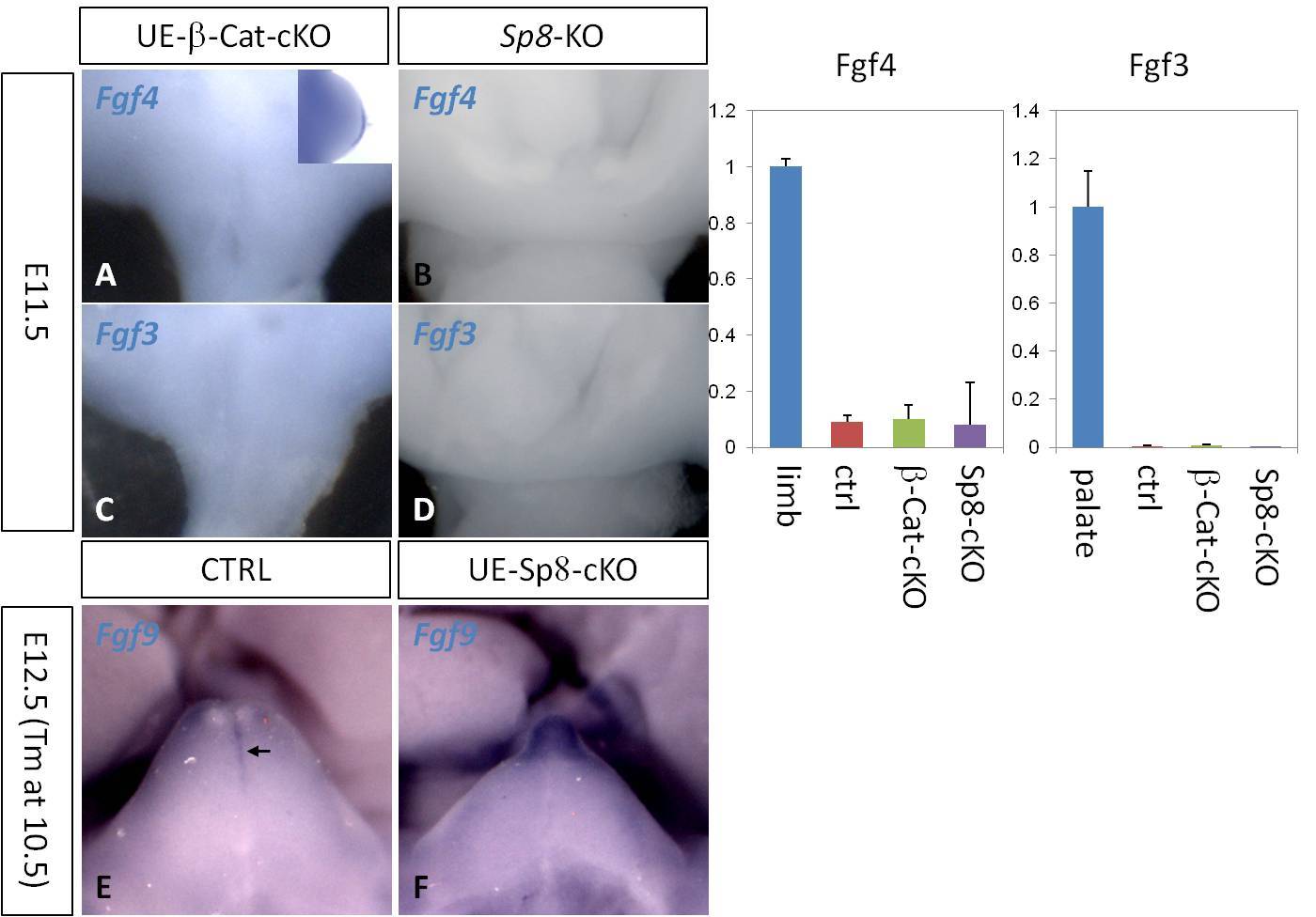

Supplement: Figure S10 — Expression of Fgfs in UE-β-Cat- and Sp8-cKOs (A–F) In situ hybridization using probes indicated. Note no Fgf4 (A–B) or Fgf3 (C–D) expression was detected in either mutants (A positive control for the Fgf4 in situ is shown in the inset of A), and downregulation of dUE Fgf9 expression (arrow in E) in Sp8-cKO (F). (JPG) [file pgen.1003231.s010.jpg]

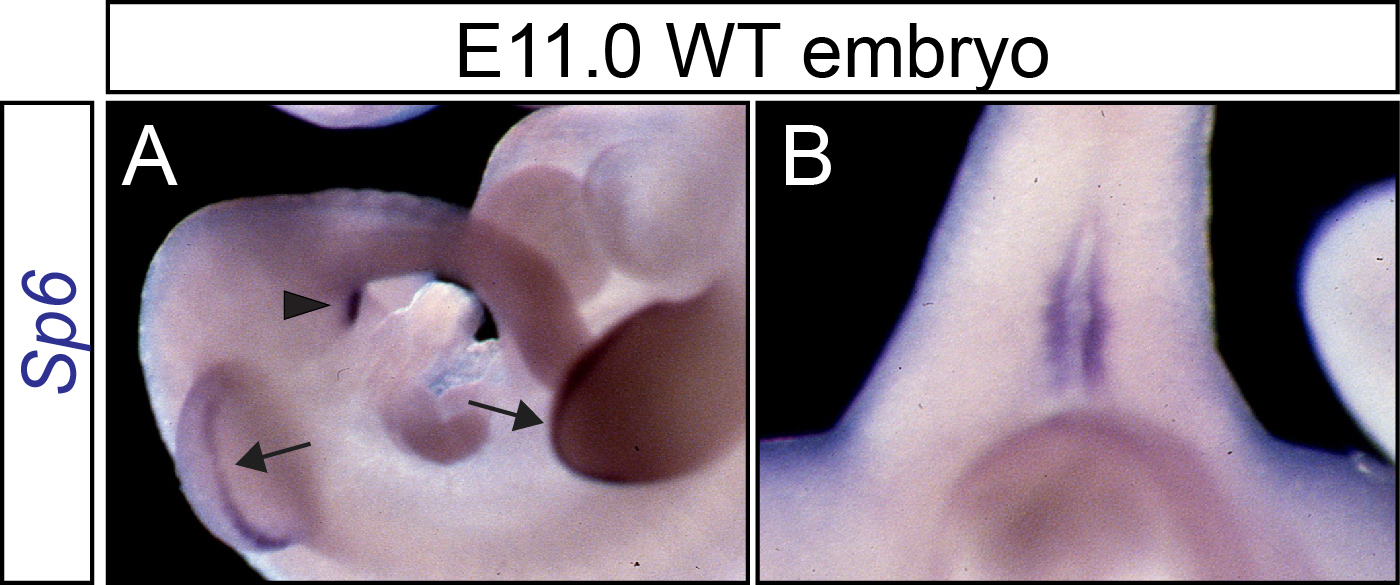

Supplement: Figure S11 — Sp6 expression in E11.0 WT embryos. (A, B) Whole mount Sp6 in situ showing AER expression (arrows in A) and UE expression (B). (JPG) [file pgen.1003231.s011.jpg]
